# A cross-sectional study on the nasopharyngeal microbiota of individuals with SARS-CoV-2 infection across three COVID-19 waves in India

**DOI:** 10.3389/fmicb.2023.1238829

**Published:** 2023-09-06

**Authors:** Tungadri Bose, Wasimuddin, Varnali Acharya, Nishal Kumar Pinna, Harrisham Kaur, Manish Ranjan, Jandhyala SaiKrishna, Tulasi Nagabandi, Binuja Varma, Karthik Bharadwaj Tallapaka, Divya Tej Sowpati, Mohammed Monzoorul Haque, Anirban Dutta, Archana Bharadwaj Siva, Sharmila S. Mande

**Affiliations:** ^1^TCS Research, Tata Consultancy Services Limited, Pune, Maharashtra, India; ^2^Centre for Cellular and Molecular Biology (CSIR-CCMB), Hyderabad, Telangana, India; ^3^TCS Genomics Lab, Tata Consultancy Services Limited, Noida, Uttar Pradesh, India

**Keywords:** nasopharyngeal microbiome, COVID-19 disease, SARS-CoV-2, variants of concern, Indian cohort, 16S rRNA gene amplicon-based sequencing

## Abstract

**Background:**

Multiple variants of the SARS-CoV-2 virus have plagued the world through successive waves of infection over the past three years. Independent research groups across geographies have shown that the microbiome composition in COVID-19 positive patients (CP) differs from that of COVID-19 negative individuals (CN). However, these observations were based on limited-sized sample-sets collected primarily from the early days of the pandemic. Here, we study the nasopharyngeal microbiota in COVID-19 patients, wherein the samples have been collected across the three COVID-19 waves witnessed in India, which were driven by different variants of concern.

**Methods:**

The nasopharyngeal swabs were collected from 589 subjects providing samples for diagnostics purposes at the Centre for Cellular and Molecular Biology (CSIR-CCMB), Hyderabad, India and subjected to 16s rRNA gene amplicon - based sequencing.

**Findings:**

We found variations in the microbiota of symptomatic vs. asymptomatic COVID-19 patients. CP showed a marked shift in the microbial diversity and composition compared to CN, in a wave-dependent manner. Rickettsiaceae was the only family that was noted to be consistently depleted in CP samples across the waves. The genera *Staphylococcus*, *Anhydrobacter*, *Thermus*, and *Aerococcus* were observed to be highly abundant in the symptomatic CP patients when compared to the asymptomatic group. In general, we observed a decrease in the burden of opportunistic pathogens in the host microbiota during the later waves of infection.

**Interpretation:**

To our knowledge, this is the first analytical cross-sectional study of this scale, which was designed to understand the relation between the evolving nature of the virus and the changes in the human nasopharyngeal microbiota. Although no clear signatures were observed, this study shall pave the way for a better understanding of the disease pathophysiology and help gather preliminary evidence on whether interventions to the host microbiota can help in better protection or faster recovery.

## Introduction

Severe Acute Respiratory Syndrome-Coronavirus 2 (SARS-CoV-2) is the causative agent for the pandemic Coronavirus Disease 2019 where there have been 768,983,095 confirmed cases of Coronavirus disease 2019 (COVID-19), including 69,53,743 deaths as of 2^nd^ August 2023 [WHO Coronavirus (COVID-19) Dashboard | WHO Coronavirus (COVID-19) Dashboard With Vaccination Data]. Since its onset, multiple variants of the virus have been reported, some of which have been categorized as variants of concern (VOC) *viz.* Alpha, Beta, Gamma, Delta, and Omicron (Aleem et al., 2022). While certain factors such as comorbidities, gender and age influence the onset of the disease as well as the severity of symptoms (Abu-Hammad et al., 2020), the effect of the SARS-CoV-2 variants on the severity of COVID-19 disease is also speculated. Differences in transmissibility and entry pathways of the variants (Menni et al., 2022; Zhao et al., 2022) could have potentially given rise to the spectrum of symptoms that have been observed in the affected patients across waves (Giacomelli et al., 2020; Ludvigsson, 2020; Menni et al., 2022). Most patients showed signs of acute respiratory distress syndrome and typical symptoms such as fever, dry cough and tiredness (*Coronavirus disease (COVID-19) [who.int]*) while other groups suffered from pain, anosmia, nasal congestion, sore throat and diarrhea (Khatiwada and Subedi, 2020). Moreover, a majority of the population was asymptomatic of the viral infection thus, acting as a hidden carrier (Day, 2020).

**Figure 1:**
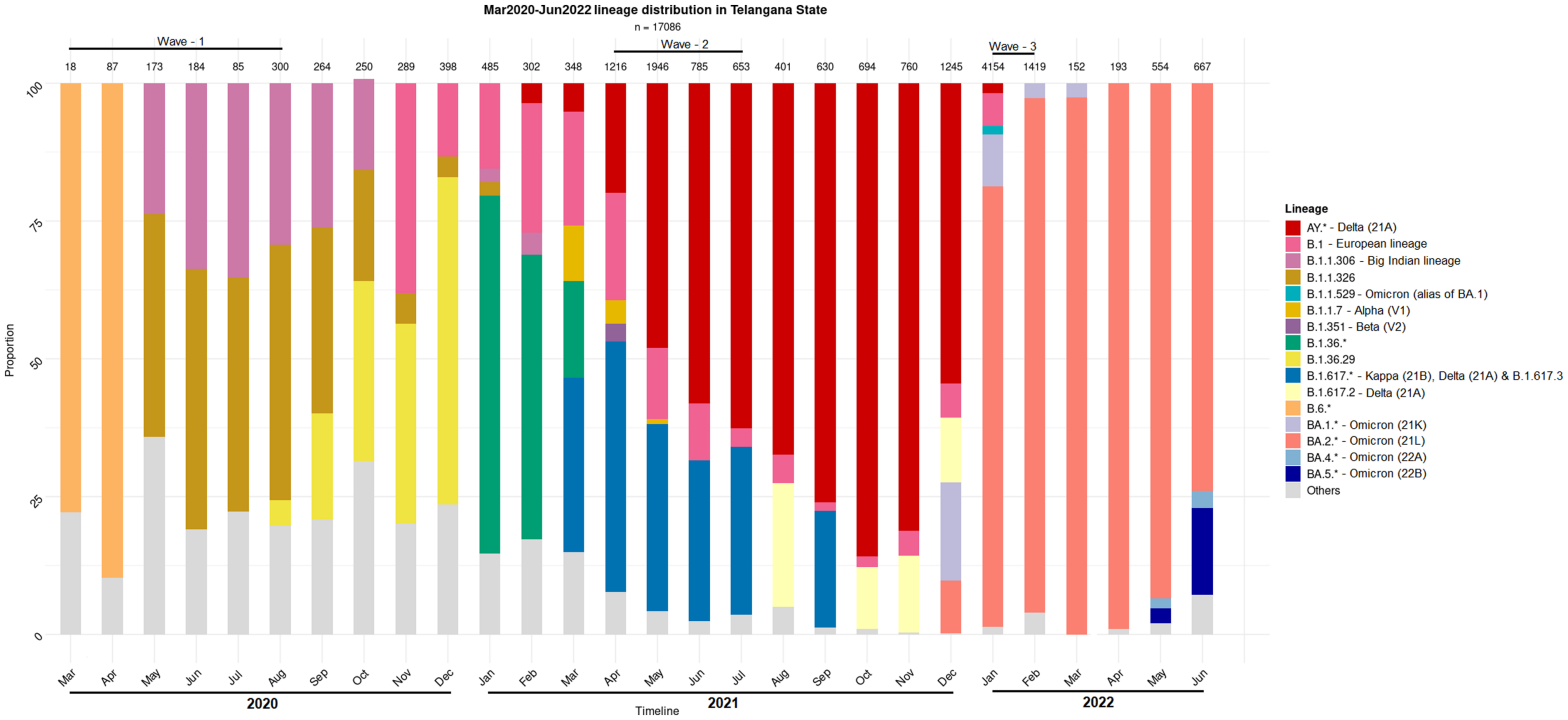
Distribution of the SARS-CoV-2 lineage among the samples collected from COVID-19 positive individuals. The nasopharyngeal samples were collected in the state of Telangana (India) between March 2020 and June 2022.

Human microbiota plays a significant role in modulating the host health by forging the immune system and it is also well known that the dysbiosis of the same has implications for diseases (Yeoh et al., 2021; Zhang et al., 2021; Wang B. et al., 2022). Therefore, association of the resident microbiota (or a dysbiosis thereof) with the symptoms and severity of the COVID-19 disease becomes worth exploring in context of different variants of concern. While some prominent studies in this direction have focused on gut microbiome dysbiosis in the COVID-19 infected patients (Zuo et al., 2020, 2021; Ng et al., 2022); recent reports also discuss the dynamics and the alterations of the nasopharyngeal microbiome (Mostafa et al., 2020; Hoque et al., 2021; Kumar et al., 2021). However, these studies have mostly been restricted to the early waves of the pandemic, and do not include microbiome profiling during subsequent waves, which were caused by distinct viral variants (Figure 1) and wherein the disease presentations were also different (first wave fuelled by variant A2, second wave by Delta variant and third wave by the Omicron variant) (Braun et al., 2021; Ventero et al., 2021; Tchoupou Saha et al., 2022). Considering the strong association between viral and bacterial co-infections and respiratory disease severity, understanding the nasopharyngeal niche microbiome composition in context of the virulence/infectivity of different SARS-CoV-2 variants becomes imperative.

SARS-CoV-2 primarily enters the human body through Angiotensin Converting Enzyme 2 (ACE-2) and Transmembrane serine protease 2 (TMPRSS2) receptors present on the alveolar epithelial cells, and nasal epithelial cells of the nasopharyngeal tract and gradually moves toward the lungs (Hoffmann et al., 2020; Ou et al., 2021). The delta and omicron variants have been observed to utilize different entry pathways for infection (Menni et al., 2022; Zhao et al., 2022), and it is likely that during this migration, the resident microbiota can get altered and any inclusion of the pathobionts may aggravate the disease outcome. Studies by independent researchers have reported that nasopharyngeal microbiota during COVID-19 is characterized by a general decrease in the abundance of commensal organisms along with an increase in the abundance of opportunistic pathogens (Liu et al., 2021; Nardelli et al., 2021; Shi et al., 2022). However, the individual organisms, both commensal as well as opportunistic, reported in each study seemed to vary. While this difference could be attributed to the choice of experimental protocols; geographical/ethnic differences between the subjects involved in each of the studies as well as the relatively lower number of samples analyzed in these studies, they are also expected to play a major role in the study outcome. For instance, the opportunistic pathogens identified to be associated with the COVID-19 microbiota in an Indian study (Gupta et al., 2022) differed considerably from those reported in studies conducted in other geographies (Mostafa et al., 2020; Liu et al., 2021; Shi et al., 2022). Consequently, it is crucial to understand the nasopharyngeal microbiota signature of COVID-19 patients in India in the context of variants as well as waves through study of a larger cohort.

With the above objectives in mind, human nasopharyngeal microbiota was profiled from Indian subjects. The samples were provided for diagnostics and sequencing of SARS-CoV-2 at Centre for Cellular and Molecular Biology (CSIR-CCMB), Hyderabad, India. The samples collected from the subjects/patients were grouped into four categories based on COVID-19 infection status (positive or negative) as well as symptom presentation (symptomatic or asymptomatic), and further processed for high throughput 16S rRNA gene amplicon-based sequencing for microbiome profiling. In addition to uncovering the variations between microbial signatures of COVID-19 positive patients (CP) versus COVID-19 negative individuals (CN), the study also reports how the nasopharyngeal microbiota changed during the pandemic over the three waves in India.

## Materials and methods

### Sample collection

This study was conducted for all three waves of COVID-19 in India, from March 2020 to February 2022 in accordance with the guidelines of the Indian Council of Medical Research (ICMR), Government of India and approved by the Institutional Ethics Committee of Centre for Cellular and Molecular Biology (CSIR-CCMB) [IEC-83/2020]. A total of 646 nasopharyngeal swab samples, were collected from individual subjects of both sex, between age groups 25–50, from different districts of Telangana state in India (data from 589 samples was used for further analysis after discarding 57 samples for various reasons including poor sequencing quality, depth, etc.). The samples corresponded to the three COVID-19 waves in India and the time periods for swab collection for each wave were as follows: March 2020 to August 2020 for the first wave, April 2021 to July 2021 for the second, and January 2022 to February 2022 for the third. The swab samples were stored at −80°C until nucleic acid extraction, to ensure DNA quality. Subjects from each wave were divided into COVID-19 positive (CP) and COVID-19 negative (CN) groups. Among the CP group, subjects reporting severe symptoms such as fever, dry/wet cough, nasal congestion, sore throat, body ache, tiredness, breathlessness, etc. were further categorized as symptomatic COVID-19 positive (sCP) and CP without symptoms were denoted as asymptomatic COVID-19 positive (aCP). The CN group were further divided into asymptomatic (aCN, apparently healthy controls) and symptomatic (sCN), i.e., COVID-19 negative subjects with presumably respiratory/other infections.

The samples used in this study were received for SARS-CoV-2 diagnostics and genome sequencing at CSIR-CCMB, also an ICMR-approved COVID-19 testing center. The diagnosis of COVID-19 patients involved combining the results from the real-time reverse transcription-polymerase chain reaction (qRT-PCR) assay performed on nasopharyngeal swabs, at the BSL-2 facility at CSIR-CCMB.

### DNA isolation

DNA was extracted from the viral transport media (VTM) containing the nasopharyngeal swabs using the QIAamp^®^ DNA Microbiome Kit (QIAGEN, Hilden, Germany) according to the manufacturer’s instructions. All the extraction procedures were performed in the pre-PCR designated room in the Biosafety Level – 2 (BSL-2) facility. The yield of the extracted DNA was validated by Nanodrop Spectrophotometric (Thermofisher Scientific, Milan, Italy) reading at 260/280 as well as 260/230 and the quality was assessed by running an aliquot of the DNA in a 1% agarose gel.

### 16S rRNA gene amplification and sequencing

The first round of Polymerase Chain Reaction (PCR) was performed in a 20 μL reaction mixture, with 50 ng of bacterial genomic DNA as the template. The primer pair targeting the V4 hypervariable region (515-F and 806-R) (Caporaso et al., 2011) of the 16S rRNA gene was modified by adding the Illumina overhang forward and reverse adaptor sequences (indicated in brackets below).

The primer pair targeting the V4 hypervariable region (515-F and 806-R)

**V4 515-F**

(TCGTCGGCAGCGTCAGATGTGTATAAGAGACAG)**GTGCC AGCMGCCGCGGTAA**

**V4 806-R**

(GTCTCGTGGGCTCGGAGATGTGTATAAGAGACAG)**GGA CTACHVGGGTWTCTAAT**

The component concentration of each reaction mixture included 10 μL of 2X EmeraldAmp^®^ GT PCR master mix (Takara Bio, Japan), 1 μL (0.5 μM) of each primer, and 7 μL of Nuclease free water. Thermocycling was performed on a Bio-Rad T100 Thermal cycler and included an initial denaturation at 95°C for 3 min, followed by 35 cycles of 95°C for 30s, 55°C for 30s, and 72°C for 30s, followed by a final extension of 72°C for 5 min. Each PCR reaction mixture was loaded into a 2% agarose gel, stained with ethidium bromide to observe the amplification.

The second round of PCR involved attaching dual indices and Illumina sequencing barcodes to the purified 16S rRNA gene amplicons, which was performed using the Nextera XT Index kit as mentioned in the manufacturer’s protocol. The prepared metagenomic libraries were then quantified and normalized using Qubit dsDNA BR Assay before getting pooled in equimolar concentrations. Our constructed libraries were then sequenced (seven sequencing runs, Supplementary Table S1) in a paired-end mode (2 × 300) on the Illumina MiSeq sequencing platform using v3 600 cycles reagent.

### Bioinformatics analysis

#### Data QC and ASV generation

A series of bioinformatics steps were followed to ensure usage of high-quality reads for the generation of the Amplicon Sequence Variants (ASVs, i.e., sequence differing by as little as one nucleotide). The raw reads were processed using cutadapt (version 3.4) prior to their analysis through the DADA2 pipeline (version 1.20) for ASV generation and subsequent taxonomic assignment. Based on the quality of reads generated across the seven sequencing runs, the forward and reverse read sequences were trimmed to 170 and 140 bases, respectively in the filterAndTrim step of DADA2. Furthermore, all read pairs with non-standard nucleotide bases, more than two ‘expected errors’ (maxEE), and lengths lower than 100 bases were discarded. Reads (and their pairs) which encountered atleast one base called with a quality score ≤ 8 (truncQ = 8) were also discarded. To account for the run-based biases, the ASVs were generated separately for each of the runs. The error models for each of the runs in the learnErrors step of DADA2 was performed using the parameters randomize = T and nbase = 5e+8. To aid in faster computation, the denoising step (wherein the error model was employed for denoising) was preceded by a dereplication step (derepFastq). The initial sequences table generated for each of the seven sequencing runs (through the makeSequenceTable step of DADA2) were finally merged using mergeSequenceTables command of DADA2. Next, the removeBimeraDenovo step (method = “consensus”) was employed to remove ASVs originating from chimeric sequences. The retained ASVs were annotated using the assignTaxonomy function in DADA2 following the naïve Bayesian classifier method with the silva_nr_v132 database. Further, the species for these ASVs were assigned using addSpecies function with the silva_species_assignment_v132 database. As a final quality control step, ASVs with extremely low counts (n < 10 across all the sequenced samples) and irrelevant annotations (*viz*, Order classified as ‘chloroplast’ or ‘na’; Family classified as ‘mitochondria’ or ‘na’; Phylum classified as ‘uncharacterized’ or ‘na’) were also removed. The final microbiota data consisted of 8,645 ASVs belonging to 589 samples and were used for subsequent analysis.

#### Alpha diversity

To determine intra individual diversity, we calculated two alpha diversity measures: observed number of ASVs (observed ASVs) and Shannon diversity using the Phyloseq package (*phyloseq*, 1.26.1). Using generalized linear mixed models (GLMMs), we modeled alpha diversity according to COVID-19 status (CN; *n* = 285, CP; *n* = 304), wave (First; *n* = 181, Second; *n* = 217, Third; *n* = 191), interaction between them (COVID-19*Wave), symptoms (Asymptomatic [aCP + aCN]; *n* = 361, Symptomatic [sCP + sCN]; *n* = 228), and sequencing depth to account for differential sequencing effort between samples, sequencing run and to control the random effect of batch processing of samples (*lme4*). In addition, we included the variables age group (25–29y; *n* = 149, 30–34y; *n* = 141, 35–39y; *n* = 122, 40–44y; *n* = 82, 45–50y; *n* = 95), gender (female; *n* = 276, male; *n* = 313) and Ct-value as these factors could influence the microbiome. To facilitate model convergence, sequencing depth was scaled and to control the run effect we included run as a random factor variable. We used a log distribution for modeling the count data (observed ASVs) and a normal distribution for modeling the continuous data (Shannon diversity). Model selection was based on the information-theoretic (IT) approach using a second-order Akaike’s information criterion corrected for small sample sizes (AIC_C_) as an information criterion and Akaike weights (ω) to determine model support. For all GLMMs, we report both conditional and marginal coefficients of determination of each model (R^2^_GLMM(c)_, which explains the variance of both the fixed and random factors, and R^2^_GLMM(m)_, which explains the variance of the fixed factors only), which we calculated as the variance explained by the best model and the ΔAIC_C_. Finally, we performed Tukey’s HSD test to detect differences between each individual category (i.e., COVID-19*Wave) on the above performed GLMMs’ outcome.

#### Beta diversity

To assess the nasopharyngeal bacterial community composition between individuals, we calculated the Jaccard and Bray–Curtis distance matrices using the Phyloseq package (*phyloseq*, 1.26.1). Jaccard accounts for presence-absence of the taxa whereas Bray-Curtis in addition gives weight to taxa abundance. We tested for differences in microbial beta diversity for COVID-19 in different waves using the permutational multivariate analysis of variance (PERMANOVA) test with 999 permutations implemented in the *adonis* function of the vegan package (Vegan, 2.6.2). The fixed variables in our full model were the following: symptoms, age group, gender, sequencing run, Ct-value, and sequencing depth. We retained the sequencing run variable in our full model to statistically account for its model support. To understand whether COVID-19 reflects true shift in microbial community composition or differential spread (dispersion) of data points from their group centroid, we investigated the homogeneity of the variances of COVID-19 positive and negative category using the PERMDISP test implemented in the *betadisper* function of the vegan package. To visualize patterns of separation between different sample categories, Principal Coordinates Analysis (PCoA) plots were prepared based on the Bray–Curtis dissimilarity coefficient.

#### Discriminant taxonomy analysis

To identify the differential abundant discriminating taxa between different the nasopharyngeal samples of subjects with varying COVID-19 status, symptoms and across different waves; negative binomial Wald tests were performed using the DESeq2 package (v 1.34.0). For this analysis, only 2,120 (out of 8,645) ASVs which were present in at least two samples were considered. In order to obtain differentially abundant taxa between CP and CN groups after correcting for the wave effect (i.e., overall) Design-1 (design = ~ Wave + Covid) was adopted for DESeq2 analysis. Further, Design-2 (design = ~ Wave + Covid + Wave:Covid) was implemented to understand the individual and combined effects (interaction) of wave (Wave) and COVID-19 status (Covid) on the microbiota composition. In addition, to ascertain the effect of symptoms (sym_asym) in conjugation with COVID-19 status (Covid), Design-3 (design = ~ sym_asym + Covid + sym_asym:Covid) was used. For each of the designs, DESeq2 was performed with default parameters except for the size factor estimation. The size factor estimation was done using “poscounts.” The discriminating taxonomies were also analyzed for higher taxonomic levels. For this purpose, the ASVs were also aggregated at higher taxonomic levels to generate abundance tables corresponding to Phyla, Family and Genus and a protocol similar to the ASV level analysis was followed. To obtain the significant taxonomic groups across the comparisons log2FoldChange and lfcSE (log2FoldChange Standard Error) were plotted using the ggplot (v 3.3.5) package in R environment (R version 4.1.1, R Development Core Team, 2011) software.

#### Network construction and identification of driver taxa

Correlational networks were constructed from the microbial abundance data to understand associations among the interacting taxa. All analyses were performed at the Genus level to have a dataset with a reasonable number of nodes for a good display and meaningful interpretation thereof. For generating the networks, a Pearson correlation coefficient cut-off of ±0.5 was used. The networks were generated using Cytoscope (v 3.9.0) and analyzed using its ‘analyze network’ module. Further, the taxa driving the major shift in case of COVID-19 infections were identified using NetShift (Kuntal et al., 2019). The tool is hosted on https://web.rniapps.net/netshift/ and uses ‘Neighbor Shift (NESH) Score’ to identify key taxonomic groups which are more likely to drive changes from a control/ healthy, to a case/disease state.

#### Discriminant functional analysis

The functional potential of the microbiota samples was predicted using PICRUSt2 (Douglas et al., 2020). Next discriminant analysis on the inferred functions (enzymes and metabolic pathways) were performed using the DESeq2 package (v 1.34.0). A similar design as elucidated for the discriminant taxonomy analysis was adopted in this case.

## Results

### Data overview

Nasopharyngeal swab samples analyzed from 589 individuals between March 2020 – February 2022 (i.e., spanning three ‘waves of COVID-19 infection’ in India) were subjected to amplicon sequencing of the bacterial V4 hypervariable region of 16S rRNA gene on the Illumina MiSeq platform (details in Methods section). The category and wave-wise distribution of the 589 samples *viz.*, sCP, aCP, sCN, and aCN has been provided in Table 1. A total of 8,645 amplicon sequence variants (ASVs) were identified in the microbiome sequence data (Supplementary Figure S1) using the DADA2 package. Preliminary taxonomic analysis indicated the dominance of the phylum Firmicutes, Proteobacteria, and Actinobacteriota across all samples (cumulative abundance over 75%) (Supplementary Figures S2A,B). At the family level, Staphylococcaceae and Corynebacteriaceae were found to be the most abundant taxa (Supplementary Figures S2C,D).

**Table 1:** Statistics on the number of samples analyzed in the study.

Category	Covid Positives (CP)		Covid Negatives (CN)	
	With symptoms (sCP)	Without symptoms (aCP)	With symptoms (sCN)	Asymptomatic apparently healthy controls (aCN)
Total	147	157	81	204
Wave1	19	70	12	80
Wave2	81	39	60	37
Wave3	47	48	9	87

Categorization of 589 analyzed samples into sub-groups and their distribution across the three COVID-19 waves have been provided.

### Reduced microbial diversity during SARS-CoV-2 infection

**Figure 2:**
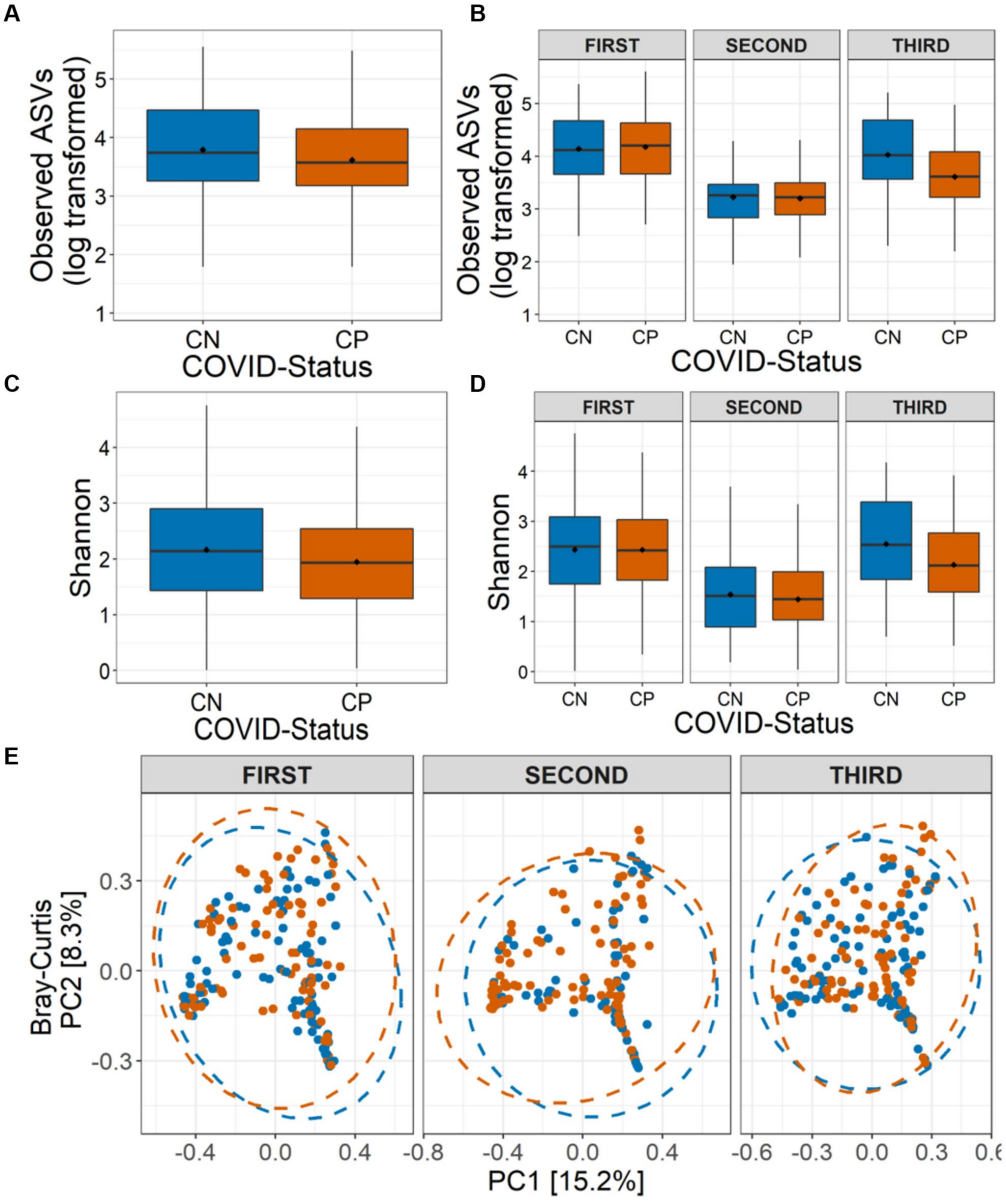
Alpha and beta diversity of the nasopharyngeal microbiota in CP and CN samples. Number of observed amplicon sequence variants (ASVs) **(A)** in all the analyzed samples (overall), and **(B)** in the three COVID-19 waves are shown. Shannon diversity in term of ASVs **(C)** in all the analyzed samples, and **(D)** in the three COVID-19 waves are depicted. **(E)** Beta diversity of microbiota assessed using principal component analysis (Bray-Curtis distance) represented along first two principal components for the three COVID-19 waves are presented.

To test whether COVID-19 status has any impact on the nasopharyngeal microbiome, we calculated the microbial alpha diversity between CP and CN samples. Using model selection based on the information-theoretic (IT) approach (Model Selection and Multimodel Inference: A Practical Information-Theoretic Approach | SpringerLink), we found strong support for an effect of COVID-19 status, wave and combined effect of COVID-19 status and wave (COVID-19*Wave) on observed ASVs (ΔAIC_C_ = 38·47, *R*^2^_GLMM(m)_ = 0·312, *R*^2^_GLMM(c)_ = 0·320, Figures 2A–B). Similarly, Shannon diversity, was observed to be influenced by COVID-19 status and Wave, individually, but not by COVID-19 status and wave together (ΔAIC_C_ = 22·85, *R*^2^_GLMM(m)_ = 0·277, *R*^2^_GLMM(c)_ = 0·312, Figures 2C–D). For both alpha diversity indices, in general, lower diversity was observed in the CP samples as compared to the CN category (Figures 2A–D). The effect of other variables included in the models such as age group, symptoms, gender, Ct-value were poorly supported by AIC_C_ model comparison for both alpha diversity indices (data not shown). Additionally, only the third wave samples showed a significantly lower diversity for observed ASVs in the CP category as compared to CN (*p* < 0·05, Tukey’s HSD) which was reflected in Shannon diversity as well (all *p* > 0·05). Overall, the results show a strong effect of COVID-19 infection on microbial alpha diversity indices, with the observed ASVs also showing an effect in a wave-dependent manner. Furthermore, the results suggest that this effect may be driven more by rare taxa than abundant ones, since the effect was less pronounced when accounting for abundance (i.e., Shannon diversity).

### Community composition perturbed in COVID-19 nasopharyngeal microbiome

We also assessed the influence of COVID-19 status on the nasopharyngeal microbial community composition. While sequencing run (Jaccard: *R*^2^ = 0·010, *p* = 0·002; Bray-Curtis: *R*^2^ = 0·011, *p* = 0·004) and sequencing depth (Jaccard: *R*^2^ = 0·011, *p* = 0·001; Bray-Curtis: *R*^2^ = 0·016, *p* = 0·001) did impact the community compositions, controlling for these factors, the PERMANOVA models indicated a strong support for, the influence of COVID-19 status (Jaccard: *R*^2^ = 0·003, *p* = 0·006; Bray-Curtis: *R*^2^ = 0·003, *p* = 0·007), wave (Jaccard: *R*^2^ = 0·026, *p* = 0·001; Bray-Curtis: *R*^2^ = 0·037, *p* = 0·001) and the interaction between them (COVID-19*Wave) (Jaccard: *R*^2^ = 0·005, *p* = 0·001; Bray-Curtis: *R*^2^ = 0·006, *p* = 0·002) on the nasopharyngeal microbial beta diversity estimates (Figure 2E). Additionally, Ct-value (Jaccard: *R*^2^ = 0·003, *p* = 0·005; Bray-Curtis: *R*^2^ = 0·003, *p* = 0·004) and symptoms (Jaccard: *R*^2^ = 0·002, *p* = 0·034; Bray-Curtis: *R*^2^ = 0·002, *p* = 0·017) also influenced the community composition. However, no effect of age group or gender (all *p* > 0·05) on the nasopharyngeal microbial community composition was observed. PERMDISP^1^ tests showed a true shift in microbial community composition and no dispersion effect (all *p* > 0·05) (Figure 2E).

### Microbes associated with disease status across different COVID-19 waves

Given the observed variation of microbial taxonomic diversity across three COVID-19 waves, negative binomial Wald tests were performed using the DESeq2 package with the intent to identify the taxa that are differentially abundant between CP and CN groups corresponding to different waves. While there were no significant differences (BH corrected *p* < 0·05) in the first wave samples, there was a marked compositional variation in the CP microbiota of the second and third wave. Cyanobacteria and Firmicutes were the dominant phyla, with Planctomycetota, Deinococcota, and Bdellovibrionota having a significantly lower abundance in the CP microbiota of the second wave samples (Supplementary Figure S3). Similarly, third wave samples showed a significantly enriched proportion of Proteobacteria in CP compared to CN.

**Figure 3:**
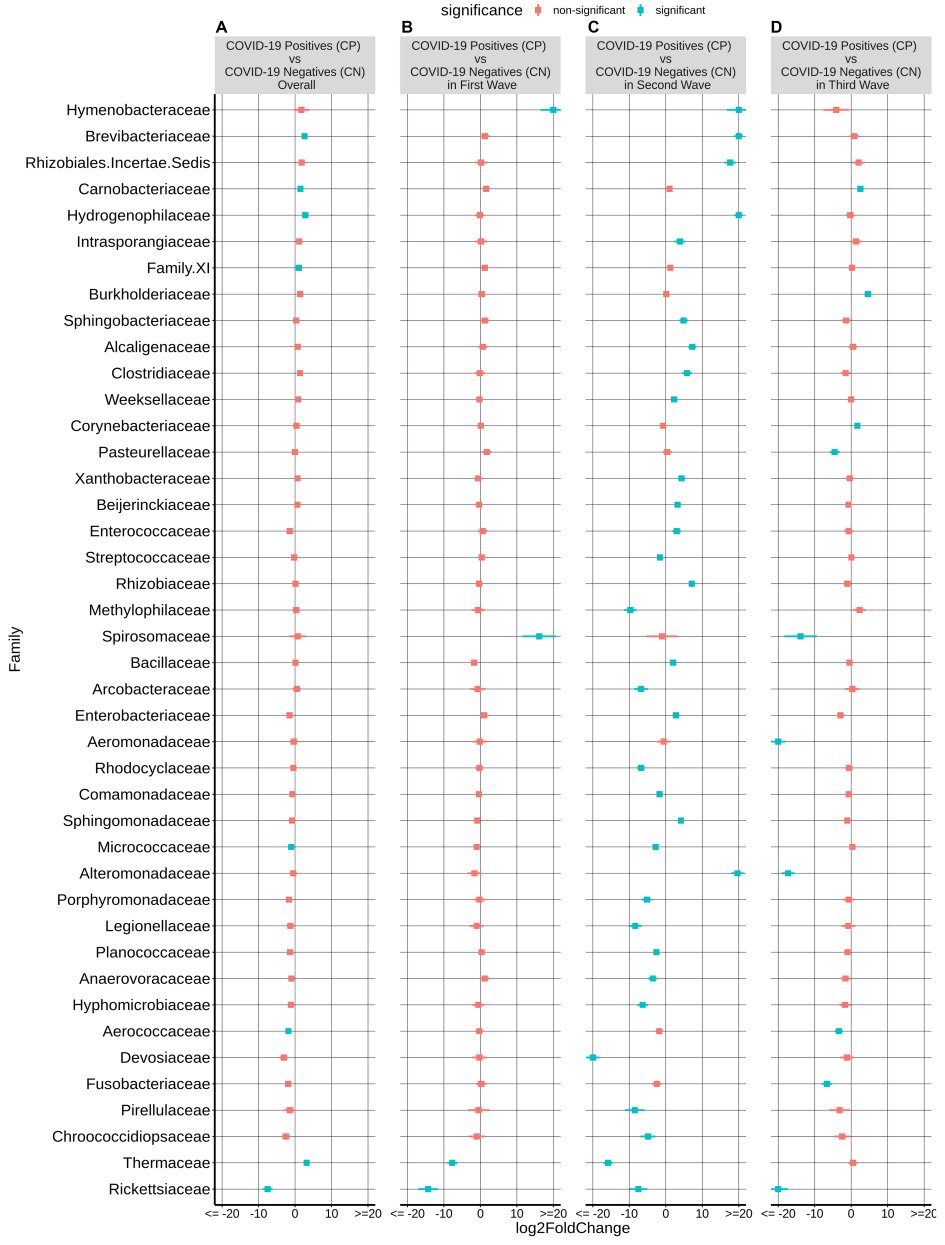
Differential abundance of bacterial families between the CP and CN samples. The log2fold change in the mean abundance (along with whiskers representing standard errors) of a bacterial family in CP with respect to CN is depicted in **(A)** all the analyzed samples (overall) as well as in **(B–D)** each of the three COVID-19 waves. Significantly different abundance (*q*-value <0.05) is indicated with blue color.

At the family level, among the taxonomic groups discriminating between CP and CN in each of the three waves, Rickettsiaceae was found to be depleted in all the three waves as seen in Figure 3. A small number of taxa showed significant differences between CP and CN microbiota in the first wave (enriched abundance of Hymenobacteraceae and Spirosomaceae with a decrease in the abundance of Thermaceae and Rickettsiaceae in CP). Additionally, the CP group of second wave samples were enriched with five bacterial families (Rhizobiales.Incertae.Sedis, Hydrogenophillaceae, Alteromonadaceae, Hymenobacteraceae, and Brevibacteriacceae) while two of them (Devosiaceae and Thermaceae) had a lower abundance. In the case of the third wave, three families (*viz.* Carnobacteriaceae, Burkholderiaceae, and Corynebacteriaceae) had a higher abundance whereas seven families (*viz.* Aeromonadaceae, Rickettsiaceae, Alteromonadaceae, Pasteurellaceae, Spirosomaceae, Aerococcaceae, Fusobacteriaceae) exhibited a lower abundance in the CP group. Furthermore, at the genera and ASV levels, the genera *Aliterella* and ASVs: ASV36 (family Rickettsiaceae), ASV161 (*Gemella*), ASV195 (*Deinococcus*), and ASV431 (*Elizabethkingia*) were noted to demonstrate a significant decrease in abundance in the CP group across all the three infection waves (Supplementary Table S2).

### Microbes associated with inter-wave variations in CP and CN groups

Most CP cases particularly during the second and the third COVID-19 wave in India were dominated by one of the viral variants (Figure 1). Supplementary Figure S4 shows the effect of different waves (and thus viral variant) on the bacterial taxonomic proportions observed in CN and CP groups of samples in terms of fold changes at the phylum level. As expected, based on the observations from the alpha and beta diversity tests, the microbiome of the second wave samples was distinct from the other two. When compared to the first wave, the second wave samples were found to be significantly enriched with the phyla Proteobacteria, Campylobacterota and Firmicutes, in both CP and CN groups. In contrast, Cyanobacteria, Planctomycetota, Bdellovibrionota, and Fusobacteriota were depleted in both the CP and CN samples from the second wave when compared to those from the first wave. When the third wave samples were compared with those from the second wave, the phyla Cyanobacteria, Plantomycetota, and Bdellovibrionota were significantly enriched in the third wave in both CP and CN samples. On the other hand, only Firmicutes were observed to be significantly depleted in the CP and CN samples of the third wave when compared to the second wave. The first wave and the third wave samples were observed to be more similar to each other. The only significant differences pertained to enrichment of Deinococcota and depletion of Bacteriodota in the CP and CN samples of the third wave compared to the first wave samples. It was intriguing to note that the phylum Deinococcota showed a gradual (and in most cases significant) increasing trend over the three waves in both CP and CN groups.

Additional results depicting the wave effect on microbiota at genera and ASV levels are provided in Table 2. Among the CP group, the genera *Abiotrophia*, *Streptococcus*, *Rheinheimera* along with *X.Eubacterium*.brachy.group and *Escherichia. Shigella* were found to follow a constant decrease in abundance between the corresponding waves (i.e., these genera were most abundant in the first wave CP samples and their abundances were least in the third wave CP samples). In the CN group, *Schlegelella* had a significantly increasing trend, whereas Acinetobacter and *Megasphaera* showed a significant decreasing pattern. Supplementary Table S2 shows a list of the ASVs and genera which followed a consistent pattern of significant increase or decrease in abundance in the CP and CN groups.

**Table 2:** List of the amplicon sequence variants (ASVs) and genera demonstrating a consistent increase or decrease in abundance across the three COVID-19 waves.

COVID-19 Positive group									
ASV/Genus	Base Mean	Wave 3 vs. Wave 2		Wave 2 vs. Wave 1		Phylum	Family	Genus	Species
		log2 Fold change	padj	log2 Fold change	padj				
ASV22	2229.73	36.39	0	4.96	0.004	Proteobacteria	Burkholderiaceae	*Burkholderia-Caballeronia-Paraburkholderia*	#N/A
ASV54	218.86	−23.34	0	−4.34	0.014	Firmicutes	Veillonellaceae	*Veillonella*	*parvula*
ASV90	94.61	−31.89	0	−6.1	0.008	Actinobacteriota	Micrococcaceae	*Rothia*	*mucilaginosa*
ASV79	63.9	−23.75	0	−7.47	0.001	Firmicutes	Streptococcaceae	*Streptococcus*	*anginosus*
ASV97	61.34	−22.32	0	−8.11	0	Firmicutes	Streptococcaceae	*Streptococcus*	*anginosus*
ASV80	51.09	−20.14	0	−8.6	0	Bacteroidota	Porphyromonadaceae	*Porphyromonas*	*pasteri*
ASV53	45.31	−29.74	0	−6.78	0	Proteobacteria	Moraxellaceae	*Acinetobacter*	*baumannii*
ASV135	38.35	−24	0	−5.91	0.005	Firmicutes	Anaerovoracaceae	*[Eubacterium] brachy group*	#N/A
ASV78	21.02	−15.59	0	−10.35	0	Proteobacteria	Rhizobiales Incertae Sedis	*Phreatobacter*	#N/A
ASV206	19.01	−22.96	0	−5.67	0.006	Firmicutes	Streptococcaceae	*Streptococcus*	#N/A
ASV255	17.98	−27.66	0	−8.86	0.001	Firmicutes	Lachnospiraceae	#N/A	#N/A
ASV330	17.36	−21.87	0	−6.18	0.019	Firmicutes	Lachnospiraceae	*Oribacterium*	*parvum*
ASV186	14.78	−34.09	0	−6.58	0.002	Proteobacteria	Xanthobacteraceae	*Afipia*	#N/A
ASV345	13.48	−21.39	0	−6.74	0.001	Firmicutes	Anaerovoracaceae	*[Eubacterium] nodatum group*	#N/A
ASV420	11.7	−29.13	0	−5.59	0.042	Firmicutes	Veillonellaceae	*Veillonella*	#N/A
ASV143	8.3	−21.98	0	−5.72	0.007	Proteobacteria	Pasteurellaceae	*Haemophilus*	#N/A
ASV37	7.7	−17.19	0	−8.1	0.003	Firmicutes	Streptococcaceae	*Streptococcus*	anginosus
*Streptococcus*	3262.93	−1.94	0.015	−1.57	0.042	Firmicutes	Streptococcaceae	*Streptococcus*	#N/A
*X.Eubacterium.brachy.group*	16.22	−19.19	0	−8.88	0	#N/A	#N/A	#N/A	#N/A
*Abiotrophia*	14.95	−20.77	0	−6.44	0.001	Firmicutes	Aerococcaceae	*Abiotrophia*	#N/A
*Rheinheimera*	8.46	−34.41	0	−5.01	0.011	Proteobacteria	Alteromonadaceae	*Rheinheimera*	#N/A
*Escherichia.Shigella*	5.5	−37.8	0	−5.31	0.005	#N/A	#N/A	#N/A	#N/A

COVID-19 Negative group									
ASV/Genus	base Mean	Wave 3 vs. Wave 2		Wave 2 vs. Wave 1		Phylum	Family	Genus	Species
		log2 Fold Change	padj	log2 Fold Change	padj				
ASV24	96.08	−16.68	0	−11.19	0	Firmicutes	Streptococcaceae	*Streptococcus*	*mitis*
ASV207	47.7	4.79	0	3.77	0.012	Proteobacteria	Comamonadaceae	*Schlegelella*	*aquatica*
ASV53	45.31	−23.72	0	−4.61	0.001	Proteobacteria	Moraxellaceae	*Acinetobacter*	*baumannii*
ASV134	11.26	−20.36	0	−6.61	0.037	Firmicutes	Lachnospiraceae	*Lachnoanaerobaculum*	*orale*
ASV404	6.98	−17.56	0	−7.66	0.01	Firmicutes	Veillonellaceae	*Megasphaera*	*micronuciformis*
*Acinetobacter*	146.43	−2.6	0.002	−2.28	0.011	Proteobacteria	Moraxellaceae	*Acinetobacter*	#N/A
*Schlegelella*	31.81	4.3	0.001	3.33	0.015	Proteobacteria	Comamonadaceae	*Schlegelella*	#N/A
*Megasphaera*	7.33	−17.02	0	−8.89	0	Firmicutes	Veillonellaceae	*Megasphaera*	#N/A

Significant changes having an adjusted value of *p* <0.05 have been reported.

### Microbial taxa associated with symptomatic and asymptomatic COVID-19 positive patients

Supplementary Figure S5 shows the overall bacterial taxonomic proportions observed in CN and CP groups of samples categorized based on symptom in terms of fold changes at the phylum levels (abundance plots are seen in Supplementary Figures S2B,D). Within the CP group, sCP had a significant overabundance of the phyla Camphylobacterota, Patescibacteria, and Firmicutes, while showing a depletion in the abundance of the phyla Fusobacteria and Proteobacteria compared to the asymptomatics (aCP).

**Figure 4:**
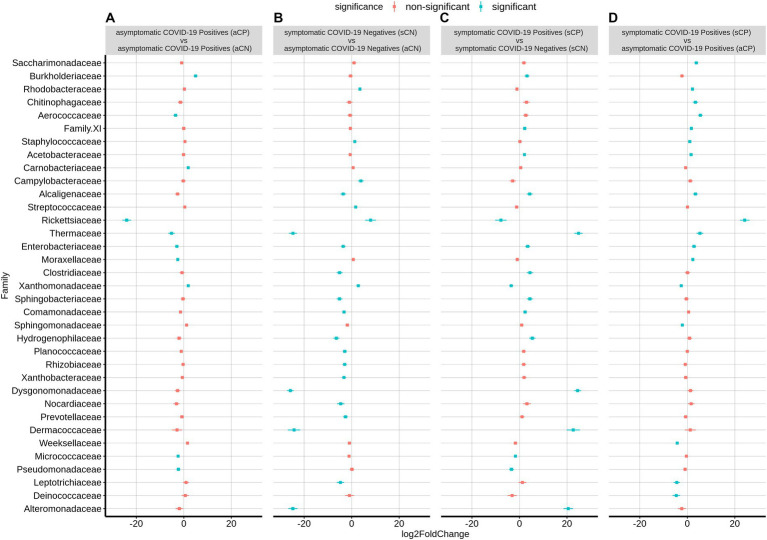
Differential abundance of bacterial families between the four sub-group of samples – aCP, sCP, aCN, and sCN. The log2fold change in the mean abundance (along with whiskers representing standard errors) of a bacterial family in **(A)** aCP with respect to aCN, **(B)** sCN with respect to aCN, **(C)** sCP with respect to sCN, and **(D)** sCP with respect to aCP is depicted. Significantly different abundance (*q*-value <0.05) is indicated with blue color.

At the family level, sCP was observed to have a significantly higher abundance of Rickettsiaceae, Aerococcaceae, and Thermaceae among others as well as a significantly lower abundance of Leptotrichiaceae, Weeksellaceae, Deinococcaceae, Sphingomonadaceae, and Xanthobacteraceae (the latter two belonging to phylum Proteobacteria) when compared to aCP (Figure 4). With respect to the microbiota samples from aCN subgroup, the signature of the aCP microbiota was characterized by a reduced abundance of Rickettsiaceae, Enterobacteriaceae (both belonging to phylum Proteobacteria), Moraxellaceae, Pseudomonadaceae (both belonging to phyla Pseudomonadota), Aerococcaceae, Thermaceae, and Micrococcaceae. At the same time, an enriched abundance of Burkholderiaceae, Xanthobacteraceae (both Proteobacteria) and Carnobacteriaceae were observed in aCP. Further, within the symptomatic subgroups, Thermaceae, Dysgonomonadaceae, Dermacoccaceae, and Alteromonadaceae were among the bacterial families with significant enrichments in sCP (w.r.t. sCN). In contrast, w.r.t. sCN, Rickettsiaceae was observed to be the most depleted family in sCP. Notably, while the abundance of Rickettsiaceae decreased during COVID-19 infections, it has been reported to increase in other respiratory infections. Thus, it appears to be a distinguishing feature between COVID-19 and other respiratory infections. The severity of COVID-19 infection (i.e., sCP vs. aCP) appeared to be linked to a higher abundance of the family Saccharimonadaceae, and Chitinophagaceae, and a lower abundance of the family Sphingomonadaceae, and Weeksellaceae. Similarly, enrichment of Thermaceae and Enterobacteriaceae, as well as a depleted abundance of Xanthobacteraceae appeared to be markers of a healthy nasopharyngeal microbiota since this trend was observed when aCN group was compared to both the sCN and aCP groups. *Kocuria* and *Liquorilactobacillus* were also linked to a healthy nasopharyngeal microbiota (Supplementary Table S4). Further, the opportunistic pathogenic genera *Stenotrophomonas* was noted to be depleted in a healthy respiratory tract microbiota. It was interesting to find that in comparison to the aCN group, the microbiota of the sCN group was seen to be enriched with *Staphylococcus*, *Stenotrophomonas* and *Campylobacter* among others. These organisms are known to possess pathogenic properties and may be responsible for the symptoms exhibited by the patients of the sCN group.

The list of the significant changes in abundance of microbes at a genus level between sCP and aCP have been provided in Table 3. *Thermus, Aerococcus*, *Enhydrobacter, and Staphylococcus* were found to be the most significantly enriched genera in sCP when compared to aCP. ASV108 (*Thermus amyloliquefaciens*) was found to be enriched in sCP in comparison to both aCP (Supplementary Table S4) indicating that its levels in the nasopharyngeal microbiota might act as an early indicator of the severity of the disease outcome. In contrast, *Elizabethkingia, Leptotrichia*, and *Veillonella* were more abundant in aCP (w.r.t. sCP) (Table 3).

**Table 3:** List of the bacterial genera which showed significant changes in abundance between sCP and aCP sub-groups.

Genus	Base Mean	log2 Fold change	padj	Phylum	Family	Genus
Elizabethkingia	89.341	−4.8943	0.0019	Bacteroidota	Weeksellaceae	Elizabethkingia
Leptotrichia	49.941	−4.8144	0.0085	Fusobacteriota	Leptotrichiaceae	Leptotrichia
Veillonella	245.31	−2.6977	0.0145	Firmicutes	Veillonellaceae	Veillonella
Staphylococcus	54,139	1.2499	0.003	Firmicutes	Staphylococcaceae	Staphylococcus
Enhydrobacter	161.48	2.8906	0.0411	Proteobacteria	Moraxellaceae	Enhydrobacter
Thermus	26.63	4.7846	0.0145	Deinococcota	Thermaceae	Thermus
Aerococcus	713.23	9.1933	3.00E-11	Firmicutes	Aerococcaceae	Aerococcus

Significant changes having an adjusted value of *p* < 0.05 have been reported. Positive log2 foldchange values indicate a higher abundance in sCP while negative log2 foldchange values represent genera with higher abundance in aCP.

### Microbial co-occurrence networks varied with COVID-19 status across the three waves

The microbial association network (Pearson correlation) corresponding to each of the three COVID-19 waves, CP, CN as well as those for symptomatic and asymptomatic conditions were constructed considering the taxonomic abundance data at the genera level. Network properties of each of these networks are provided in Table 4. In line with observations pertaining to diversity indices, minimal number of nodes (genera) and edges (interactions between nodes) were observed in the network corresponding to the second wave whereas the number of nodes and edges in the third wave network was observed to be considerably higher than that in the first two waves. The distribution of betweenness centralities of the nodes in the network seemed to indicate a considerable shift in the network architecture between the subsequent waves (Supplementary Figure S6). As mentioned earlier, with respect to the first wave, we noted a loss of many nodes in the network corresponding to the second wave, including the genus *Actinomyces* which appeared to be a high betweenness node (degree = 14; betweenness =0·618; stress = 74) in the microbial network corresponding to the first wave (Supplementary Figure S7). The change in network architecture from the first to the second wave was driven by the genera *Cnuella*, *Marmoricola*, *Paenibacillus*, *Peptostreptococcus*, and *Solobacterium* as indicated by their NESH scores (Supplementary Table S5). Although the abundance of most of these genera (except *Cnuella*) in the second wave was lower in both CP and CN samples w.r.t. first wave (Supplementary Table S3), the driver taxa were found to disrupt the sub-network of the first wave, thereby leading to an altered association among the genera in the network representing the second wave. *Cnuella* was higher in abundance only in the CP group, while its abundance decreased in the CN group. Notably, some organisms belonging to the genera *Paenibacillus*, *Peptostreptococcus*, and *Solobacterium* are known to be opportunistic pathogens (Stein and Goldstein, 2006; Grady et al., 2016; Alauzet et al., 2021). Further, some of these driver taxonomic groups, *viz.*, *Cnuella*, *Marmoricola*, and *Paenibacillus* lost their importance in the microbiome association network corresponding to the third wave (Supplementary Figure S8). *Leptotrichia* and *Actinomyces* were noted to be taxonomic groups driving the changes during the third wave when compared to the second wave. While the abundance of *Actinomyces* decreased, the abundance of *Leptotrichia* was higher in the third wave w.r.t. the second wave (Supplementary Table S3). Further, *Leptotrichia* appeared as a node with high stress centrality (degree = 18; betweenness =0·317; stress = 236) in the third wave network.

**Table 4:** Properties of the microbial association networks analyzed in this study.

Network	No of nodes	No of edges	Avg No of neighbors	Characteristic path length	Clustering coefficient	Network density
Wave 1	85	278	5.5	1.808	0.774	0.367
Wave 2	56	92	3.33	1.4	0.667	0.667
Wave 3	92	486	4.7	2.284	0.586	0.247
Symptomatic	49	128	5.75	1.179	0.94	0.821
Asymptomatic	63	214	7.833	1.303	0.854	0.712
Covid Positive	69	214	2	3.091	0.091	0.2
Covid Negative	75	210	4.727	1.673	0.596	0.473

The microbiome association network corresponding to the symptomatic condition (considering data from all three waves as well as both CP and CN groups combined) was found to be represented by a relatively lower number of microbial groups which also interacted less coherently among themselves (average number of neighbors), compared to the network corresponding to the asymptomatic condition (Table 4). From the NetShift constructed network (Supplementary Figure S9), it was intriguing to note that the sub-network composition between the asymptomatic and symptomatic networks largely remained the same, with nodes belonging to the major four clusters showing no change in their cluster memberships. However, the betweenness centralities of some of these nodes changed between the symptomatic and the asymptomatic condition, indicating a change in the overall network architecture (Supplementary Figure S9). Such changes were most evident in the clusters 2 and 3. Among other noticeable changes, the genera *Methyloversatilis* seemed to gain prominence (both an increased NESH score as well as a higher abundance) in the network representing the symptomatic condition (Supplementary Table S5). In contrast *Porphyromonas*, *Campylobacter*, and *Skermanella* were found to lose their importance in the third wave network due to a decrease in their abundance or that of their interacting partners (Supplementary Table S5; Supplementary Figure S9).

**Figure 5:**
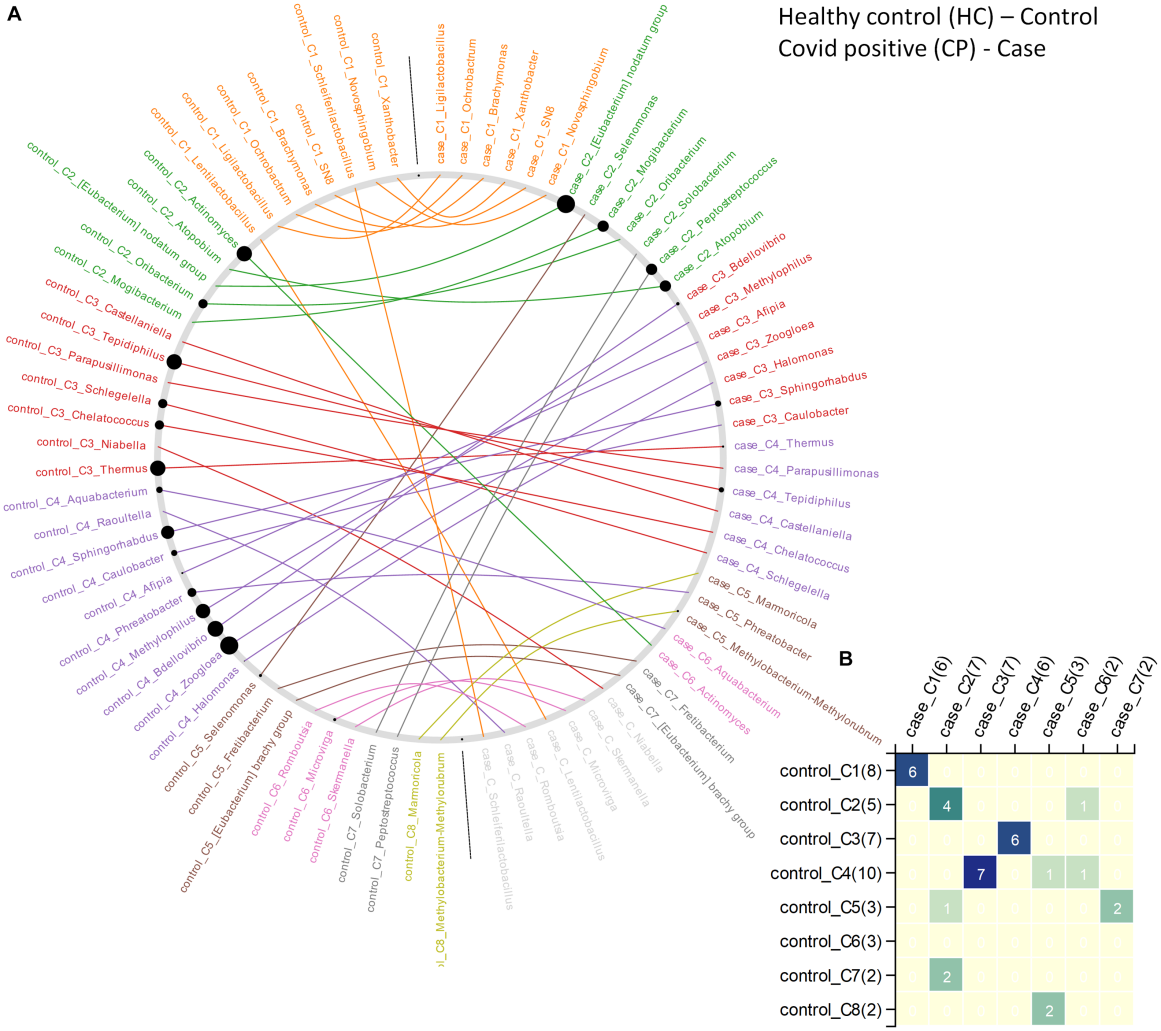
The changes in community structure (community shuffling) between the microbial association networks corresponding to the CN – ‘control’ and CP – ‘case’ networks. **(A)** Nodes belonging to the CN – ‘control’ and CP – ‘case’ networks are plotted along the left half and right half of the circular frame. Same node (microbe) in the two network is connected by an edge for easy viewing of the community shuffling. Node labels are colored (at random) based on sub-network/community affiliations. Grayed out node labels indicate that the node does not interact directly with the common sub-network. The node sizes are proportional to the betweenness centrality measure of the node in the corresponding network. **(B)** Heatmap representing the communities in the CN – ‘control’ network (along the vertical axis) and the CP – ‘case’ network (along the horizontal axis) with counts of the nodes (microbes) in each community in brackets. Numbers (and color gradient) in the heatmap indicates how the nodes constituting any sub-network/community of the CN – ‘control’ network are shared with the sub-networks/communities of the CP – ‘case’ network, and vice versa.

In case of CP and CN networks, the CP-network was characterized by longer path lengths (3·091) compared to the CN-network (1·3). The network density values indicate a denser CN-network compared to the CP-network. The number of nodes with high betweenness values were also higher in the CN network compared to the CP network. The interacting partners for the microbes were seen to considerably change from the CN to CP. This resulted in the generation of alternate microbial sub - networks between the two conditions which might have an implication on the behavior of the individual microbes (Figures 5A,B). Major changes were observed in clusters 2, 3, and 4 of the CN networks. Several members of cluster 2, such as *[Eubacterium] nodatum* group, *Atopobium*, and *Mogibacterium* were characterized by an increased NESH score in the CP network. Notably, the genera *Schlegelella* which was earlier found to consistently increase in abundance across the three waves of the COVID-19 pandemic in the CN samples (Table 2), witnessed a drop in its betweenness centrality measure in the CP network, thereby indicating its strong association with the CN nasopharyngeal microbiota (Figure 5).

### Association of inferred functions with microbiota samples during COVID-19

Across the three COVID-19 waves, the metabolic pathway for methanogenesis from acetate (METH-ACETATE-PWY) was found to be significantly enriched in the microbiota corresponding to the CP samples Supplementary Table S6. In contrast, the pathways for anaerobic gondoate biosynthesis (PWY-7663), aerobic respiration I using cytochrome c (PWY-3781), cis-vaccenate biosynthesis (PWY-5973), Kdo transfer to lipid IVA III (PWY-6467), and chlorophyllide a biosynthesis I (CHLOROPHYLL-SYN) appeared to be depleted functions in the CP group as inferred using PICRUSt2. While IgA-specific metalloendopeptidase (EC:3.4.24.13) appeared to be the most enriched enzyme in the CP group, sedolisin (EC:3.4.21.100) was most enriched in the CN group (Supplementary Table S6).

In terms of the functional differences between the CP and CN groups in each of the three COVID-19 waves, while the minimum number of differentially abundant pathways were seen among the first wave samples, the quantum of change among the discriminating pathways was relatively smaller (log2foldchange < ±2) in the third wave samples (Supplementary Table S6). Further, there was no overlap between the metabolic pathways differentially expressed between CP and CN samples in the first two COVID-19 waves with those in the third COVID-19 wave. In the first two COVID-19 waves, spirilloxanthin and 2,2′-diketo-spirilloxanthin biosynthesis pathway (PWY-6581) and coumarins biosynthesis pathway (PWY-7398) were seen to be enriched in the CP group. In contrast, the depleted pathways in the CP group (w.r.t. CN group) included Kdo transfer to lipid IVA III pathway (PWY-6467), L-methionine biosynthesis III pathway (HSERMETANA-PWY), TCA cycle V and VI pathways (PWY-6969 and PWY-5913), aerobic respiration I pathway (PWY-3781), coenzyme B biosynthesis pathway (P241-PWY), and NAD biosynthesis I (from aspartate) pathway (PYRIDNUCSYN-PWY). Notably, of the pathways which were found to be depleted in CP samples from the first two COVID-19 waves, L-methionine biosynthesis III pathway (HSERMETANA-PWY) and TCA cycle V and VI pathways (PWY-6969 and PWY-5913) were found to be marginally enriched in the CP samples from the third COVID-19 wave.

Supplementary Tables S8, S9 provide the list of functional pathways and enzymes which were found to be differentially enriched among the four disease categories. 4-hydroxyphenylacetate degradation (3-HYDROXYPHENYLACETATE-DEGRADATION-PWY), and 3-phenylpropanoate degradation (P281-PWY) appeared to be enriched in the microbiota of the CP samples when compared to CN irrespective of symptoms.

Irrespective of COVID-19 disease status, the nasopharyngeal microbiota of the symptomatic patients, i.e., sCP and sCN were seen to be enriched for the following functional pathways w.r.t. the asymptomatic patients – pyrimidine deoxyribonucleotide phosphorylation (PWY-7197), (aerobic) heme biosynthesis I (HEME-BIOSYNTHESIS-II), *de novo* biosynthesis II of adenosine (PWY-7220) and guanosine (PWY-7222) deoxyribonucleotides, and pyrimidine nucleobases salvage (PWY-7208). The sucrose degradation IV pathway (PWY-5384) was, however, noted to be enriched only in sCN (when compared to aCN) but depleted in sCP (when compared to aCP). In contrast, the superpathway of mycolyl-arabinogalactan-peptidoglycan complex biosynthesis (PWY-6404) was found to be enriched in CP (when compared to CN) only in the symptomatic sub-group. Conversely, the pathways for sucrose degradation IV (PWY-5384), O-antigen building blocks biosynthesis (OANTIGEN-PWY), and mixed acid fermentation (FERMENTATION-PWY) were noted to be enriched in the aCP sub-group while compared to aCN.

## Discussion

The present study aimed at profiling the nasopharyngeal microbiota of Indian subjects, across the three COVID-19 waves, to identify signatures, if any, which may be distinct between the microbiota sample collected from CP and CN individuals. It may be noted that the different infection waves were caused by different viral variants. In particular, the second and the third COVID-19 wave was predominated by the Delta (21A) 334 Omicron (21L) variants, respectively, (Figure 1). Given that subtle variations exist in the pathophysiology of different viral variants (Menni et al., 2022; Zhao et al., 2022) it was anticipated that the associated host microbiota would also exhibit certain changes in each of the three waves.

Irrespective of the disease status, the nasopharyngeal microbiota in Indian samples was found to be dominated by the phylum Firmicutes, Proteobacteria, and Actinobacteriota and the family Staphylococcaceae and Corynebacteriaceae. This is in line with the reports presented in earlier studies inspecting nasopharyngeal microbiota in other geographies (Allen et al., 2014; Crovetto et al., 2022; Giugliano et al., 2022). However, we noted that the proportion of Firmicutes was two to three times higher than Proteobacteria in the nasopharyngeal microbiota in Indian samples, especially in the CN group. This concurs with the observations reported in earlier studies from the US and Spain (Allen et al., 2014; Crovetto et al., 2022), while is in contrast to those observed in Italians wherein Proteobacteria was found to be the most dominant organism (Giugliano et al., 2022).

Our findings indicate decreased microbial diversity associated with COVID-19 infections. Earlier studies too have shown that the diversity of the microbes constituting the nasopharyngeal microbiota decreased in patients with confirmed COVID-19 infections (Mostafa et al., 2020; Giugliano et al., 2022; Gupta et al., 2022) barring a few exceptions (Crovetto et al., 2022). A study by Gupta A et al. on a relatively smaller cohort in India had further identified host type (age and gender) and patient condition (symptomatic vs. asymptomatic) as potential factors affecting the enrichment of specific bacterial communities in upper respiratory tract (Gupta et al., 2022). In our study, while the condition of the patients (i.e., symptomatic vs. asymptomatic) was found to be associated with bacterial diversity alone; while age and gender did not seem to significantly influence the same (Supplementary Figure S5). The conscious choice of collecting samples from patients/subjects of a narrow age range of 25–50 years (adults expected to possess strong immunity) is the likely cause of age not impacting the enrichment of specific bacterial communities in our study. Additionally, we also saw that the diversity of the nasopharyngeal microbiota varied across the three waves (Figure 2; Supplementary Figure S2). While we expected such changes given the behavioral changes adopted during COVID-19 pandemic, we were more intrigued to find that the microbes which were found to be distinguishing between CP and CN were also varied across the waves in most cases. For instance, while the abundances of Cyanobacteria, Firmicutes, Planctomycetota, Deinococcota, and Bdellovibrionota significantly differed between the CP and CN groups in the second-wave, Proteobacteria was significantly different between the third-wave CP and CN samples (Supplementary Figure S5).

It may be noted that the depletion of Rickettsiaceae in CP samples was the only consistent pattern that was observed across the three COVID-19 waves at a family level. At an ASV level, this observation was supported by a significant drop in the abundance of ASV36 in CP w.r.t. CN (Table 2). Notably, while the abundance of Rickettsiaceae decreased during COVID-19 infections, it has been reported to increase in other respiratory infections (McGinn and Lamason, 2021). Thus, it appears to be a distinguishing feature between COVID-19 and other respiratory infections. To our knowledge, this is the first report on the (negative) association of the abundance of Rickettsiaceae with CP. Further studies into this aspect will be required to see if the presence of Rickettsiaceae in the nasopharyngeal microbiota could provide any advantage in combating the onset/progression of a COVID-19 infection.

Other consistent patterns across the three COVID-19 waves included depletion of the genera *Aliterella* as well as certain ASVs from the genera *Deinococcus* (ASV195) and *Elizabethkingia* (ASV431) in CP. This is consistent with a previous study involving SARS-CoV-2 infected patients which have also shown the absence of microbes belonging to the phylum Deinococcus-Thermus in patients admitted to ICU (Rueca et al., 2021). It was intriguing to note that despite multiple reports on the risk and incidences of septicemia caused by *Elizabethkingia* in COVID-19 patients (Das et al., 2022; Ong et al., 2022) the abundance of *Elizabethkingia* was found to be lower in CP compared to CN. While the causation of septicemia in most case has been attributed to *E. meningoseptica* (Das et al., 2022; Ong et al., 2022) the ASV corresponding to *Elizabethkingia* (ASV431) identified in the current study matched closer to *E. anopheles* (with 100% query coverage and 99·6% sequence identity) when compared to *E. meningoseptica* (having 100% query coverage and 98·42% sequence identity). Given an earlier report on the contamination of throat swab collection kits with *E. anopheles* we presume that the observed enrichment of *Elizabethkingia* in CN could also be an artifact.

In contrast, *Gemella massiliensis* (ASV161) was enriched in CP across all the three waves. Interestingly there have been contradictory reports regarding the association of *Gemella* with COVID-19 infection. While studies conducted in China had reported a depletion of *Gemella* species like *G. morbillorum* and *G. haemolysans* in swab samples collected from the pharynx of COVID-19 patients (Shi et al., 2022) a study involving subjects in India had shown an increment in the abundance of *Gemella* to be associated with CP (Gupta et al., 2022). It is therefore likely that the association of *Gemella* with COVID-19 infection might vary across geographies. The observation pertaining to a lower abundance of genera *Aliterella* in CP across all the three COVID-19 waves was also noteworthy. While none of the previous literature on association of microbiota with COVID-19 reported a similar observation, a few works exploring the antiviral properties of Cyanobacteria drew our attention (Barre et al., 2021; Sami et al., 2021). Cyanobacteria such as *Aliterella* are a rich source of bioactive compounds and are likely to possess antiviral properties which supports their higher abundance in the CN group.

From the perspective of the potential role of microbiota in the manifestation of COVID-19 infection, our study reports the enrichment of IgA-specific metallo-endopeptidase enzyme (EC:3.4.24.13) in CP samples (Supplementary Table S7–overall). This metal-dependent enzyme cleaves the Pro-Thr bond in the hinge region of the heavy chain in immunoglobulin A (IgA) and is known to be encoded by pathogenic bacterial groups like *Streptococcus* (Kornfeld and Plaut, 1981; Gilbert et al., 1991). Given the role of IgA in mucosal immunity (Chao et al., 2020) and the reports on the association of IgA with the criticality of COVID-19 disease (Zervou et al., 2021), it appears that CP patients are indeed vulnerable to secondary bacterial infections.

Given the observed variations in the nasopharyngeal microbiota across the COVID-19 waves (Figures 2, 3; Supplementary Figures S1–S3), we were interested to find which of the changes in the microbiota followed similar trends in both the CP and CN groups. The microbiota abundance signature (at a phylum level) of the first and the third wave seemed to be similar with each other as compared to that of the second wave (Supplementary Figures S2, S3). Across both the CP and CN groups, the abundance of the phylum Deinococcota was found to gradually increase during the course of the pandemic (Supplementary Figure S3). Although this increase in abundance of Deinococcota was not always statistically significant, the trend was very clear. A number of ASVs (and genera) were also observed to follow a consistent up/down trend in either CP or CN group across the three waves (Table 2). Most notably, in the CP group, majority of the ASVs which demonstrated a downtrend during the course of the pandemic represented opportunistic pathogens like *Veillonella parvula*, *Rothia mucilaginosa*, *Streptococcus anginosus*, *Acinetobacter baumannii*, etc. Since, previous studies have reported an increase in the abundance of opportunistic pathogens in the microbiota of COVID-19 patients compared to healthy individuals (Hoque et al., 2021; Shi et al., 2022); this observation might seem counter intuitive. It may be noted that most studies (Hoque et al., 2021; Gupta et al., 2022; Shi et al., 2022) reporting a higher abundance of opportunistic pathogens in microbiota samples from COVID-19 patients were based on data collected during the early part of the pandemic (samples collected in 2020). It is likely that during early days of the pandemic, the human immune system fighting the SARS-CoV-2 virus resulted in an immunocompromised state that could be exploited by the opportunistic pathogens to form secondary infections.

The findings by Devi et al. (2022) regarding the higher transcript abundance of *Achromobacter xylosoxidans* and *Bacillus cereus* in cases of COVID-19 associated mortality, and *Leptotrichia buccalis* in the severe COVID-19 cases highlights the role of co-infecting microbes in the severity and outcome of COVID-19. Thus, despite the observations made in our study that implicated the perturbation of nasopharyngeal microbiota as a result of the viral infection, it cannot be completely ruled out that these changes could be a potential factor aggravating the infection and disease outcome. However, during the later waves, once vaccines against the SARS-CoV-2 virus were introduced, as well as there were many more cases with prior exposure to the pathogen; it is likely that the immune system has become better equipped to deal with the challenges posed by the virus as well as to ward off secondary infections caused by opportunistic pathogens. The lower abundance of bacteria with pathogenic potential like *Streptococcus matis*, *Acinetobacter baumannii*, *Lachnoanaerobaculum orale*, and *Megasphaera micronuciformis* even in the CN group also supports the above notion (Table 2). Indeed, among the genera which were identified to drive the change in microbial network architecture from the first to the second wave (Supplementary Figure S6), several were noted to be opportunistic pathogens (such as, *Paenibacillus*, *Peptostreptococcus*, and *Solobacterium*) (Stein and Goldstein, 2006; Grady et al., 2016; Alauzet et al., 2021) whose abundance decreased in both CP and CN samples from the second wave when compared to the first wave. In addition to vaccination, lower exposure to pollutants due to lockdown and usage of masks as well as practicing home remedies for improving respiratory health might have also led to the overall decrease in the proportion of opportunistic pathogens in the nasopharyngeal tract of Indians over the course of the pandemic. An exception was noted in the case of the genera *Leptotrichia* which appeared to drive the change in the microbial network architecture (and increased in abundance) from the second to the third wave (Supplementary Figure S7). Though part of commensal respiratory microbiota, species of the genera *Leptotrichia* genera (e.g. *L. buccalis*) possess pathogenic properties and have been shown to have a higher abundance of transcripts in severe COVID-19 patients (Devi et al., 2022). In this context, it is also important to note that we did not find any bacterial function which was consistently enriched or depleted in the CP group (w.r.t. CN) across the three COVID-19 waves. As stated above, the changing nature of the underlying microbiota structure through the course of the pandemic due to vaccination, lockdown and/or other interventions might be a reason for this observation. We noted an enrichment of the pathway for chitin derivatives degradation (PWY-6906) in sCP w.r.t. sCN. While similar observations were not made in the asymptomatic group (aCP w.r.t. aCN), given the recent interest in the role of chitin derivatives as anti-viral agents (Ishihara et al., 1993; Safarzadeh et al., 2021) we thought this would be worth mentioning. This observation paves way for a deeper introspection into the microbiota’s potential in combating viral infection through the production of antiviral metabolites.

The wide variation in the symptoms in CP patients led us to investigate whether there were any microbial association with the severity of the infection, i.e., between the sCP and aCP sub-groups (sCP; *n* = 147 vs. aCP; *n* = 157). Our findings (see Table 3) differed from a recently published study by Gupta et al. which also inspected the same phenomenon using a smaller cohort of 11 symptomatic and nine asymptomatic COVID-19 positive patients (Gupta et al., 2022). While this could be due to variations in data analysis protocols, the differences in the number of samples in the two studies might have also influenced the outcome. In this context, we found a set of five ASVs (Table 5) whose abundance seemed to be linked to symptoms in response to infections in the respiratory tract. A thorough investigation into their role in disease manifestation might reveal whether and how these organisms interact with the virus and the host immune system in driving the disease outcome. Details of all the ASVs identified in this study have been provided in Supplementary Table S2. It seems pertinent to also highlight here that in the CN group, ASVs belonging to genera *Campylobacter* (high AMR/ priority) and *Streptococcus* (medium AMR/ priority) were found to be higher in the symptomatic sub-group as compared to the asymptomatic (apparently healthy) sub-group. While *Enhydrobacter* (critical AMR/ priority) and *Staphylococcus* (high AMR/priority) were enriched in the symptomatic sub-group among the CP patients.

**Table 5:** List of amplicon sequence variants (ASVs) whose abundances were associated with symptoms in response to infections in the respiratory tract.

ASV	Base Mean	sCN vs. aCN		sCP vs. aCP		Phylum	Family	Genus	Species
		log2 Fold change	padj	log2 Fold change	padj				
ASV244	6.386275	−25.7467	3.08E-10	−34.6709	2.43E-22	Actinobacteriota	Corynebacteriaceae	Corynebacterium	#N/A
ASV107	15.55177	−23.3261	3.14E-06	−35.3004	1.25E-16	Actinobacteriota	Corynebacteriaceae	Corynebacterium	#N/A
ASV4	579.6335	−7.21141	3.71E-05	−4.9978	0.009012	Actinobacteriota	Corynebacteriaceae	Corynebacterium	#N/A
ASV10	148.3991	−3.87589	0.029283	−5.78461	0.000129	Bacteroidota	Weeksellaceae	Elizabethkingia	anophelis/bruuniana/meningoseptica/miricola/occulta/ursingii
ASV36	206.5742	10.64582	0.000916	24.7539	1.37E-21	Proteobacteria	Rickettsiaceae	#N/A	#N/A

Significant changes having an adjusted value of *p* <0.05 have been reported. Positive log2 foldchange values indicate a higher abundance of the ASV in symptomatic samples, and vice versa.

In addition, we were also intrigued to find the enrichment of superpathway of mycolyl-arabinogalactan-peptidoglycan (mAGP) complex biosynthesis (PWY-6404) in the sCP when compared to sCN sub-group. Since mAGP complex is best known to be associated with the viability of *Mycobacterium tuberculosis*, we speculate that symptomatic COVID-19 positive (sCP) patients have a likelihood to activate latent tuberculosis infection. Some of the recent reports also support the notion (Sereda et al., 2022; Wang Q. et al., 2022). With the rapid enhancement in microbiome research, it has become evident that the manifestation of a viral infection in the human body is an outcome of a complex interplay between the host, the virus, and the resident microbiota (Yi et al., 2014; Yeoh et al., 2021).

It may be noted that the samples for the study were obtained from the patients and their relatives presenting themselves at the testing centers for COVID-19 diagnosis. As a result, the number of samples for a few of the sub-categories were relatively low (see Table 1), thereby preventing finer statistical comparisons involving them. For the same reasons, only the most relevant information (metadata) could be obtained from the subjects. Additional metadata, such fate of the patient, vaccination status, re-infections or co-morbidities if any, medication regime, etc. if available, could have been helpful in inferring more robust clinical correlations.

## Conclusion

Given the expected variations in the host microbiota across different regions in the world, the present study aimed at presenting the changes in the nasopharyngeal microbiota in COVID-19 positive individuals in an Indian context. The study design also focused on identifying the variations in the microbiota signatures between the symptomatic and asymptomatic individuals and present a set of microbes which might play a role in minimizing the symptoms of a respiratory infection. Furthermore, most studies on the effect of COVID-19 infection on the human nasopharyngeal microbiota were conducted during the early period of the pandemic. Reports capturing the effect of the changing nature of the virus and/or interventions like vaccination, isolation (due to lockdown) on the host microbiota are largely missing. Our study, possibly for the first time, presents the change in the human nasopharyngeal microbiota over the course of the pandemic, which had varied presentation in all the three waves in India, largely caused by different SARS-CoV-2 variants (spanning the first two years). Through examining the nasopharyngeal microbiota of COVID-19 positive as well as COVID-19 negative Indians, our study reports both the consistent and the varying patterns in the microbiota across the three COVID-19 waves, in terms of microbial diversity, taxonomy and inferred functions. Our study reveals a perturbation in the microbial diversity and composition with a gradual drop in the abundance of opportunistic pathogens in the human respiratory tract across both the CP and CN groups over the course of the pandemic. Whether and how the above mentioned changes assist in COVID-19 disease onset and/or progression, would be interesting to explore through further studies. Overall, the findings are expected to enhance the general understanding of the human nasopharyngeal microbiota and aid in devising strategies to ward off other respiratory pathogens in the future.

## Data availability statement

The datasets presented in this study can be found in online repositories. The names of the repository/repositories and accession number(s) can be found below: https://www.ncbi.nlm.nih.gov/, bioproject PRJNA902495.

## Ethics statement

The studies involving humans were approved by Institutional Ethics Committee of CSIR – Centre for Cellular and Molecular Biology [IEC – 83/2020]. The studies were conducted in accordance with the local legislation and institutional requirements. The human samples used in this study were acquired from samples received for SARS-CoV-2 diagnostics and genome sequencing at CSIR-CCMB, also an ICMR-approved COVID-19 testing center. Written informed consent for participation was not required from the participants or the participants’ legal guardians/next of kin in accordance with the national legislation and institutional requirements.

## Author contributions

MMH and SSM conceived the idea. ABS, AD, BV, DTS, KBT, MMH, SSM, and TB created the study design. ABS, BV, KBT, and DTS designed the sequencing protocol. VA and TN performed the wet lab, including sequencing experiments. W, HK, MR, NKP, TB, and JSK performed the bioinformatic analysis, ABS, AD, NKP, TB, VA, and W analyzed the results and wrote the manuscript. ABS and MMH managed the project and oversaw the overall progress. All authors read and approved the final version of the manuscript.

## Funding

The authors thank TCS CoIN (co-innovation network) program (CLP03) and SBI foundation’s CSR Grant (GAP0570) to CSIR-CCMB for supporting the microbiome sequencing experiments performed at CSIR-CCMB, Hyderabad.

## Conflict of interest

TB, NKP, HK, BV, MMH, AD, and SSM were employed by Tata Consultancy Services Limited.

The remaining authors declare that the research was conducted in the absence of any commercial or financial relationships that could be construed as a potential conflict of interest.

## Publisher’s note

All claims expressed in this article are solely those of the authors and do not necessarily represent those of their affiliated organizations, or those of the publisher, the editors and the reviewers. Any product that may be evaluated in this article, or claim that may be made by its manufacturer, is not guaranteed or endorsed by the publisher.

## Footnotes

^1^https://github.com/vegandevs/vegan

## Supplementary material

Supplementary TABLE S1 Statistics on the amplicon sequencing runs performed in this study.

Supplementary TABLE S2 List of the amplicon sequence variants (ASVs) and genera demonstrating a consistent increase or decrease in abundance across the three COVID-19 waves. Significant changes having an adjusted *p*-value < 0.05 have been reported.

Supplementary TABLE S3 Discriminatory taxonomic groups identified at a ASV and genera level between the three COVID-19 waves among samples from the CP & CN groups.

Supplementary TABLE S4 Discriminatory taxonomic groups identified at a ASV and genera level between the four sample sub-groups based on symptoms and disease status - aCP, sCP, aCN & sCN.

Supplementary TABLE S5 Table of ‘Neighbor Shift (NESH) Score’ corresponding to Figure 5 and Figures S7–S9.

Supplementary TABLE S6 Discriminatory functional pathways identified between CP & CN samples across the three COVID-19 waves using inferred functional profiles obtained through PICRUSt2 analysis.

Supplementary TABLE S7 Discriminatory enzyme functional potentials identified between CP & CN samples across the three COVID-19 waves using inferred functional profiles obtained through PICRUSt2 analysis.

Supplementary TABLE S8 Discriminatory functional pathways identified between the four sample sub-groups based on symptoms and disease status - aCP, sCP, aCN & sCN using inferred functional profiles obtained through PICRUSt2 analysis.

Supplementary TABLE S9 Discriminatory enzyme functional potentials identified between the four sample sub-groups based on symptoms and disease status - aCP, sCP, aCN & sCN, using inferred functional profiles obtained through PICRUSt2 analysis.

Supplementary Figure S1 Venn diagram representing the distribution of 8645 amplicon sequence variants (ASVs) across **(A)** four disease categories, and **(B)** three COVID-19 waves.

Supplementary Figure S2 Stacked bar-plot representing the distribution of the bacterial taxonomies across samples. Distribution of bacteria at **(A)** phylum and **(B)** family level in CP & CN samples across the three COVID-19 waves. Distribution of bacteria at **(C)** phylum and **(D)** family level between the four sample sub-groups: aCP, sCP, aCN & sCN.

Supplementary Figure S3 Differential abundance of bacterial phylum between the CP & CN samples. The log_2_fold change in the mean abundance (along with whiskers representing standard errors) of a bacterial phylum in CP with respect to CN is depicted in **(A)** all the analysed samples (overall) as well as in **(B-D)** each of the three COVID-19 waves. Significantly different abundance (q-value < 0.05) is indicated with blue colour.

Supplementary Figure S4 Differential abundance of bacterial phylum between the CP & CN samples across the three COVID-19 waves. The log_2_fold change in the mean abundance (along with whiskers representing standard errors) of a bacterial phylum in the second wave with respect to the first wave in **(A)** CN samples, **(B)** CP samples; third wave with respect to the first wave in **(C)** CN samples, **(D)** CP samples; third wave with respect to the second wave in **(E)** CN samples, **(F)** CP samples. Significantly different abundance (q-value < 0.05) is indicated with blue colour.

Supplementary Figure S5 Differential abundance of bacterial phylum between the four sub-group of samples - aCP, sCP, aCN & sCN. The log2fold change in the mean abundance (along with whiskers representing standard errors) of a bacterial phylum in **(A)** aCP with respect to aCN, **(B)** sCN with respect to aCN, **(C)** sCP with respect to sCN, and **(D)** sCP with respect to aCP is depicted. Significantly different abundance (q-value < 0.05) is indicated with blue colour.

Supplementary Figure S6 Distribution of betweenness centralities of the nodes (microbes) in each of the analysed microbial association network.

Supplementary FIGURE S7 The changes in community structure (community shuffling) between the microbial association networks corresponding to the first COVID-19 wave - ‘control’ and the second COVID-19 wave - ‘case’ networks. Nodes belonging to the ‘control’ and ‘case’ networks are plotted along the left half and right half of the circular frame. Same node (microbe) in the two network is connected by an edge for easy viewing of the community shuffling. Node labels are coloured (at random) based on sub-network/ community affiliations. Greyed out node labels indicate that the node does not interact directly with the common sub-network. The node sizes are proportional to the betweenness centrality measure of the node in the corresponding network.

Supplementary FIGURE S8 The changes in community structure (community shuffling) between the microbial association networks corresponding to the second COVID-19 wave - ‘control’ and the third COVID-19 wave - ‘case’ networks. Nodes belonging to the ‘control’ and ‘case’ networks are plotted along the left half and right half of the circular frame. Same node (microbe) in the two network is connected by an edge for easy viewing of the community shuffling. Node labels are coloured (at random) based on sub-network/ community affiliations. Greyed out node labels indicate that the node does not interact directly with the common sub-network. The node sizes are proportional to the betweenness entrality measure of the node in the corresponding network.

Supplementary FIGURE S9 The changes in community structure (community shuffling) between the microbial association networks corresponding to the samples from asymptomatic individuals - ‘control’ and the samples from symptomatic individuals - ‘case’ networks. Nodes belonging to the ‘control’ and ‘case’ networks are plotted along the left half and right half of the circular frame. Same node (microbe) in the two network is connected by an edge for easy viewing of the community shuffling. Node labels are coloured (at random) based on sub-network/ community affiliations. Greyed out node labels indicate that the node does not interact directly with the common sub-network. The node sizes are proportional to the betweenness centrality measure of the node in the corresponding network.

## References

[ref1] Abu-Hammad O. Alnazzawi A. Borzangy S. S. Abu-Hammad A. Fayad M. Saadaledin S.. (2020). Factors influencing global variations in COVID-19 cases and fatalities; a review. Healthcare (Basel) 8:216. doi: 10.3390/healthcare8030216, PMID: 32708986 PMC7551068

[ref2] Alauzet C. Aujoulat F. Lozniewski A. Ben Brahim S. Domenjod C. Enault C.. (2021). A new look at the genus Solobacterium: a retrospective analysis of twenty-seven cases of infection involving *S. moorei* and a Review of Sequence Databases and the Literature. Microorganisms 9:1229. doi: 10.3390/microorganisms9061229, PMID: 34198943 PMC8229177

[ref3] Aleem A. Akbar Samad A. B. Slenker A. K. (2022). Emerging variants of SARS-CoV-2 and novel therapeutics against coronavirus (COVID-19). Stat Pearls. Treasure Island, FL. 34033342

[ref4] Allen E. K. Koeppel A. F. Hendley J. O. Turner S. D. Winther B. Sale M. M. (2014). Characterization of the nasopharyngeal microbiota in health and during rhinovirus challenge. Microbiome 2:22. doi: 10.1186/2049-2618-2-22 25028608 PMC4098959

[ref5] Barre A. Van Damme E. J. M. Simplicien M. Le Poder S. Klonjkowski B. Benoist H.. (2021). Man-specific lectins from plants, Fungi, algae and Cyanobacteria, as potential blockers for SARS-CoV, MERS-CoV and SARS-CoV-2 (COVID-19) coronaviruses: biomedical perspectives. Cells 10:1619. doi: 10.3390/cells10071619, PMID: 34203435 PMC8305077

[ref6] Braun T. Halevi S. Hadar R. Efroni G. Glick Saar E. Keller N.. (2021). SARS-CoV-2 does not have a strong effect on the nasopharyngeal microbial composition. Sci. Rep. 11:8922. doi: 10.1038/s41598-021-88536-6 33903709 PMC8076218

[ref7] Caporaso J. G. Lauber C. L. Walters W. A. Berg-Lyons D. Lozupone C. A. Turnbaugh P. J.. (2011). Global patterns of 16S rRNA diversity at a depth of millions of sequences per sample. Proc. Natl. Acad. Sci. U. S. A. 108, 4516–4522. doi: 10.1073/pnas.1000080107 20534432 PMC3063599

[ref8] Chao Y. X. Rotzschke O. Tan E. K. (2020). The role of IgA in COVID-19. Brain Behav. Immun. 87, 182–183. doi: 10.1016/j.bbi.2020.05.057, PMID: 32454136 PMC7245198

[ref9] Crovetto F. Selma-Royo M. Crispi F. Carbonetto B. Pascal R. Larroya M.. (2022). Nasopharyngeal microbiota profiling of pregnant women with SARS-CoV-2 infection. Sci. Rep. 12:13404. doi: 10.1038/s41598-022-17542-z 35927569 PMC9352760

[ref10] Das A. Kabi S. Kar D. Sahu K. K. (2022). Prevalence of *Elizabethkingia meningoseptica* infections and their resistant pattern in tertiary care hospital. J. Pure Appl. Microbiol. 16, 967–973. doi: 10.22207/JPAM.16.2.19, PMID: 37342731

[ref11] Day M. (2020). COVID-19: four fifths of cases are asymptomatic, China figures indicate. BMJ 369:m1375. doi: 10.1136/bmj.m1375, PMID: 32241884

[ref12] Devi P. Maurya R. Mehta P. Shamim U. Yadav A. Chattopadhyay P.. (2022). Increased abundance of Achromobacter xylosoxidans and *Bacillus cereus* in upper airway transcriptionally active microbiome of COVID-19 mortality patients indicates role of co-infections in disease severity and outcome. Microbiol Spectr. 10:e0231121. doi: 10.1128/spectrum.02311-21, PMID: 35579429 PMC9241827

[ref13] Douglas G. M. Maffei V. J. Zaneveld J. R. Yurgel S. N. Brown J. R. Taylor C. M.. (2020). PICRUSt2 for prediction of metagenome functions. Nat. Biotechnol. 38, 685–688. doi: 10.1038/s41587-020-0548-6, PMID: 32483366 PMC7365738

[ref14] Giacomelli A. Pezzati L. Conti F. Bernacchia D. Siano M. Oreni L.. (2020). Self-reported olfactory and taste disorders in patients with severe acute respiratory coronavirus 2 infection: a cross-sectional study. Clin. Infect. Dis. 71, 889–890. doi: 10.1093/cid/ciaa330, PMID: 32215618 PMC7184514

[ref15] Gilbert J. V. Plaut A. G. Wright A. (1991). Analysis of the immunoglobulin a protease gene of Streptococcus sanguis. Infect. Immun. 59, 7–17. doi: 10.1128/iai.59.1.7-17.1991, PMID: 1987065 PMC257698

[ref16] Giugliano R. Sellitto A. Ferravante C. Rocco T. D'agostino Y. Alexandrova E.. (2022). NGS analysis of nasopharyngeal microbiota in SARS-CoV-2 positive patients during the first year of the pandemic in the Campania region of Italy. Microb. Pathog. 165:105506. doi: 10.1016/j.micpath.2022.105506, PMID: 35358660 PMC8958261

[ref17] Grady E. N. Macdonald J. Liu L. Richman A. Yuan Z. C. (2016). Current knowledge and perspectives of Paenibacillus: a review. Microb. Cell Factor. 15:203. doi: 10.1186/s12934-016-0603-7 PMC5134293 27905924

[ref18] Gupta A. Karyakarte R. Joshi S. Das R. Jani K. Shouche Y.. (2022). Nasopharyngeal microbiome reveals the prevalence of opportunistic pathogens in SARS-CoV-2 infected individuals and their association with host types. Microbes Infect. 24:104880. doi: 10.1016/j.micinf.2021.104880, PMID: 34425246 PMC8379005

[ref19] Hoffmann M. Kleine-Weber H. Schroeder S. Kruger N. Herrler T. Erichsen S.. (2020). SARS-CoV-2 cell entry depends on ACE2 and TMPRSS2 and is blocked by a clinically proven protease inhibitor. Cells 181:e8. doi: 10.1016/j.cell.2020.02.052 PMC7102627 32142651

[ref20] Hoque M. N. Sarkar M. M. H. Rahman M. S. Akter S. Banu T. A. Goswami B.. (2021). SARS-CoV-2 infection reduces human nasopharyngeal commensal microbiome with inclusion of pathobionts. Sci. Rep. 11:24042. doi: 10.1038/s41598-021-03245-4 34911967 PMC8674272

[ref21] Ishihara C. Yoshimatsu K. Tsuji M. Arikawa J. Saiki I. Tokura S.. (1993). Anti-viral activity of sulfated chitin derivatives against friend murine leukaemia and herpes simplex type-1 viruses. Vaccine 11, 670–674. doi: 10.1016/0264-410X(93)90315-O, PMID: 8391740

[ref22] Khatiwada S. Subedi A. (2020). Lung microbiome and coronavirus disease 2019 (COVID-19): possible link and implications. Hum Microb J. 17:100073. doi: 10.1016/j.humic.2020.100073, PMID: 32835135 PMC7405772

[ref23] Kornfeld S. J. Plaut A. G. (1981). Secretory immunity and the bacterial IgA proteases. Rev. Infect. Dis. 3, 521–534. doi: 10.1093/clinids/3.3.521 6792682

[ref1002] Kumar D. Pandit R. Sharma S. Raval J. Patel Z. Joshi M.. (2021). Nasopharyngeal microbiome of COVID-19 patients revealed a distinct bacterial profile in deceased and recovered individuals. Microb Pathog. 173, 105829–143. doi: 10.1016/j.micpath.2022.105829, PMID: 36252893 PMC9568276

[ref24] Kuntal B. K. Chandrakar P. Sadhu S. Mande S. S. (2019). 'NetShift': a methodology for understanding 'driver microbes' from healthy and disease microbiome datasets. ISME J. 13, 442–454. doi: 10.1038/s41396-018-0291-x, PMID: 30287886 PMC6331612

[ref25] Liu J. Liu S. Zhang Z. Lee X. Wu W. Huang Z.. (2021). Association between the nasopharyngeal microbiome and metabolome in patients with COVID-19. Synth Syst. Biotechnol. 6, 135–143. doi: 10.1016/j.synbio.2021.06.002, PMID: 34151035 PMC8200311

[ref26] Ludvigsson J. F. (2020). Systematic review of COVID-19 in children shows milder cases and a better prognosis than adults. Acta Paediatr. 109, 1088–1095. doi: 10.1111/apa.15270, PMID: 32202343 PMC7228328

[ref27] Mcginn J. Lamason R. L. (2021). The enigmatic biology of rickettsiae: recent advances, open questions and outlook. Pathog. Dis. 79:ftab019. doi: 10.1093/femspd/ftab019, PMID: 33784388 PMC8035066

[ref28] Menni C. Valdes A. M. Polidori L. Antonelli M. Penamakuri S. Nogal A.. (2022). Symptom prevalence, duration, and risk of hospital admission in individuals infected with SARS-CoV-2 during periods of omicron and delta variant dominance: a prospective observational study from the ZOE COVID study. Lancet 399, 1618–1624. doi: 10.1016/S0140-6736(22)00327-0, PMID: 35397851 PMC8989396

[ref29] Mostafa H. H. Fissel J. A. Fanelli B. Bergman Y. Gniazdowski V. Dadlani M.. (2020). Metagenomic next-generation sequencing of nasopharyngeal specimens collected from confirmed and suspect COVID-19 patients. MBio 11:1969. doi: 10.1128/mBio.01969-20, PMID: 33219095 PMC7686804

[ref30] Nardelli C. Gentile I. Setaro M. Di Domenico C. Pinchera B. Buonomo A. R.. (2021). Nasopharyngeal microbiome signature in COVID-19 positive patients: can we definitively get a role to *Fusobacterium periodonticum*? Front. Cell. Infect. Microbiol. 11:625581. doi: 10.3389/fcimb.2021.625581, PMID: 33659220 PMC7919745

[ref31] Ng S. C. Peng Y. Zhang L. Mok C. K. Zhao S. Li A.. (2022). Gut microbiota composition is associated with SARS-CoV-2 vaccine immunogenicity and adverse events. Gut 71, 1106–1116. doi: 10.1136/gutjnl-2021-326563, PMID: 35140064 PMC8844967

[ref32] Ong J. Y. Wang C. H. Tsai Y. S. Chen F. L. Lee C. H. Ou T. Y. (2022). Nosocomial septicemia in COVID-19 nosocomial *K. pneumoniae*, A. baumannii, and *Elizabethkingia meningoseptica* septicemia in a patient of COVID-19. J. Infect. 85, 90–122. doi: 10.1016/j.jinf.2022.04.004, PMID: 35395320 PMC8982476

[ref33] Ou T. Mou H. Zhang L. Ojha A. Choe H. Farzan M. (2021). Hydroxychloroquine-mediated inhibition of SARS-CoV-2 entry is attenuated by TMPRSS2. PLoS Pathog. 17:e1009212. doi: 10.1371/journal.ppat.1009212, PMID: 33465165 PMC7845965

[ref1003] R Core Team (2011). R: A language and environment for statistical computing.

[ref34] Rueca M. Fontana A. Bartolini B. Piselli P. Mazzarelli A. Copetti M.. (2021). Investigation of nasal/oropharyngeal microbial community of COVID-19 patients by 16S rDNA sequencing. Int. J. Environ. Res. Public Health 18:2174. doi: 10.3390/ijerph18042174, PMID: 33672177 PMC7926517

[ref35] Safarzadeh M. Sadeghi S. Azizi M. Rastegari-Pouyani M. Pouriran R. Haji Molla Hoseini M. (2021). Chitin and chitosan as tools to combat COVID-19: a triple approach. Int. J. Biol. Macromol. 183, 235–244. doi: 10.1016/j.ijbiomac.2021.04.157, PMID: 33930442 PMC8078037

[ref36] Sami N. Ahmad R. Fatma T. (2021). Exploring algae and cyanobacteria as a promising natural source of antiviral drug against SARS-CoV-2. Biom. J. 44, 54–62. doi: 10.1016/j.bj.2020.11.014, PMID: 33640332 PMC7836382

[ref37] Sereda Y. Korotych O. Klimuk D. Zhurkin D. Solodovnikova V. Grzemska M.. (2022). Tuberculosis co-infection is common in patients requiring hospitalization for COVID-19 in Belarus: mixed-methods study. Int. J. Environ. Res. Public Health 19:4370. doi: 10.3390/ijerph19074370, PMID: 35410048 PMC9028713

[ref38] Shi Y. L. He M. Z. Han M. Z. Gui H. Y. Wang P. Yu J. L.. (2022). Characterization of altered oropharyngeal microbiota in hospitalized patients with mild SARS-CoV-2 infection. Front. Cell. Infect. Microbiol. 12:824578. doi: 10.3389/fcimb.2022.824578, PMID: 35372134 PMC8965315

[ref39] Stein G. E. Goldstein E. J. (2006). Fluoroquinolones and anaerobes. Clin. Infect. Dis. 42, 1598–1607. doi: 10.1086/503907, PMID: 16652318

[ref40] Tchoupou Saha O. F. Dubourg G. Yacouba A. Bossi V. Raoult D. Lagier J. C. (2022). Profile of the nasopharyngeal microbiota affecting the clinical course in COVID-19 patients. Front. Microbiol. 13:871627. doi: 10.3389/fmicb.2022.871627, PMID: 35655997 PMC9152678

[ref41] Ventero M. P. Cuadrat R. R. C. Vidal I. Andrade B. G. N. Molina-Pardines C. Haro-Moreno J. M.. (2021). Nasopharyngeal microbial communities of patients infected with SARS-CoV-2 that developed COVID-19. Front. Microbiol. 12:637430. doi: 10.3389/fmicb.2021.637430, PMID: 33815323 PMC8010661

[ref42] Wang Q. Guo S. Wei X. Dong Q. Xu N. Li H.. (2022). Global prevalence, treatment and outcome of tuberculosis and COVID-19 coinfection: a systematic review and meta-analysis (from November 2019 to March 2021). BMJ Open 12:e059396. doi: 10.1136/bmjopen-2021-059396 PMC9213780 35725250

[ref43] Wang B. Zhang L. Wang Y. Dai T. Qin Z. Zhou F.. (2022). Alterations in microbiota of patients with COVID-19: potential mechanisms and therapeutic interventions. Signal Transduct. Target. Ther. 7:143. doi: 10.1038/s41392-022-00986-0 35487886 PMC9052735

[ref44] Yeoh Y. K. Zuo T. Lui G. C. Zhang F. Liu Q. Li A. Y.. (2021). Gut microbiota composition reflects disease severity and dysfunctional immune responses in patients with COVID-19. Gut 70, 698–706. doi: 10.1136/gutjnl-2020-323020, PMID: 33431578 PMC7804842

[ref45] Yi H. Yong D. Lee K. Cho Y. J. Chun J. (2014). Profiling bacterial community in upper respiratory tracts. BMC Infect. Dis. 14:583. doi: 10.1186/s12879-014-0583-3 25391813 PMC4236460

[ref46] Zervou F. N. Louie P. Stachel A. Zacharioudakis I. M. Ortiz-Mendez Y. Thomas K.. (2021). SARS-CoV-2 antibodies: IgA correlates with severity of disease in early COVID-19 infection. J. Med. Virol. 93, 5409–5415. doi: 10.1002/jmv.27058, PMID: 33932299 PMC8242647

[ref47] Zhang H. Ai J. W. Yang W. Zhou X. He F. Xie S.. (2021). Metatranscriptomic characterization of coronavirus disease 2019 identified a host transcriptional classifier associated with immune signaling. Clin. Infect. Dis. 73, 376–385. doi: 10.1093/cid/ciaa663, PMID: 32463434 PMC7314197

[ref48] Zhao H. Lu L. Peng Z. Chen L. L. Meng X. Zhang C.. (2022). SARS-CoV-2 omicron variant shows less efficient replication and fusion activity when compared with Delta variant in TMPRSS2-expressed cells. Emerg. Microbes Infect. 11, 277–283. doi: 10.1080/22221751.2021.2023329, PMID: 34951565 PMC8774049

[ref49] Zuo T. Liu Q. Zhang F. Lui G. C. Tso E. Y. Yeoh Y. K.. (2021). Depicting SARS-CoV-2 faecal viral activity in association with gut microbiota composition in patients with COVID-19. Gut 70, 276–284. doi: 10.1136/gutjnl-2020-322294, PMID: 32690600 PMC7385744

[ref50] Zuo T. Zhang F. Lui G. C. Y. Yeoh Y. K. Li A. Y. L. Zhan H.. (2020). Alterations in gut microbiota of patients with COVID-19 during time of hospitalization. Gastroenterology 159:e8. doi: 10.1053/j.gastro.2020.05.048 PMC7237927 32442562

