# Supplementary figures and images for: A cross-sectional study on the nasopharyngeal microbiota of individuals with SARS-CoV-2 infection across three COVID-19 waves in India
Source: Front Microbiol. 2023 Sep 6;14:1238829. doi: 10.3389/fmicb.2023.1238829 (PMC10511876; doi:10.3389/fmicb.2023.1238829)

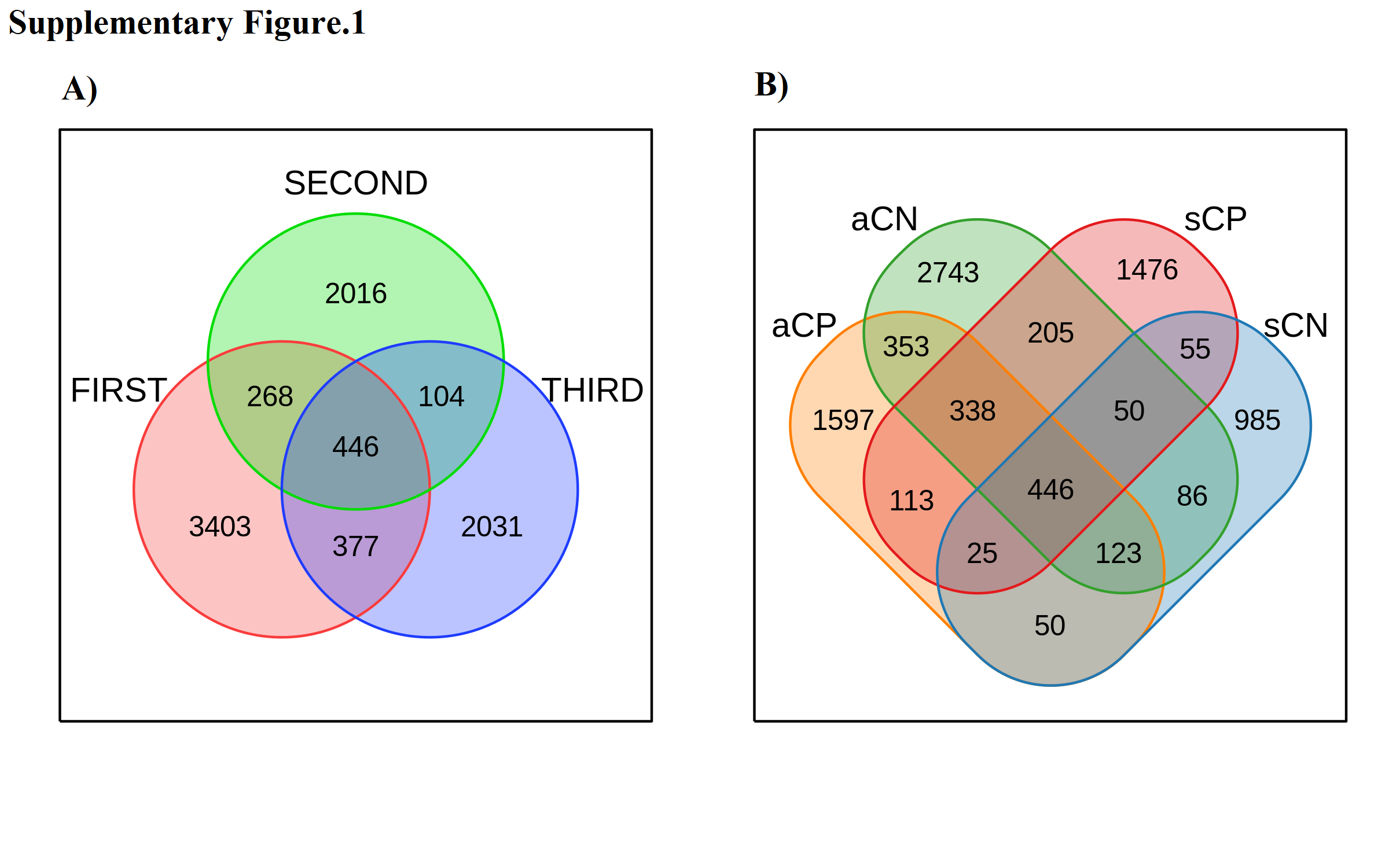

Supplement: Supplementary Figure S1 — Venn diagram representing the distribution of 8645 amplicon sequence variants (ASVs) across (A) four disease categories, and (B) three COVID-19 waves. [file Image_1.TIFF]

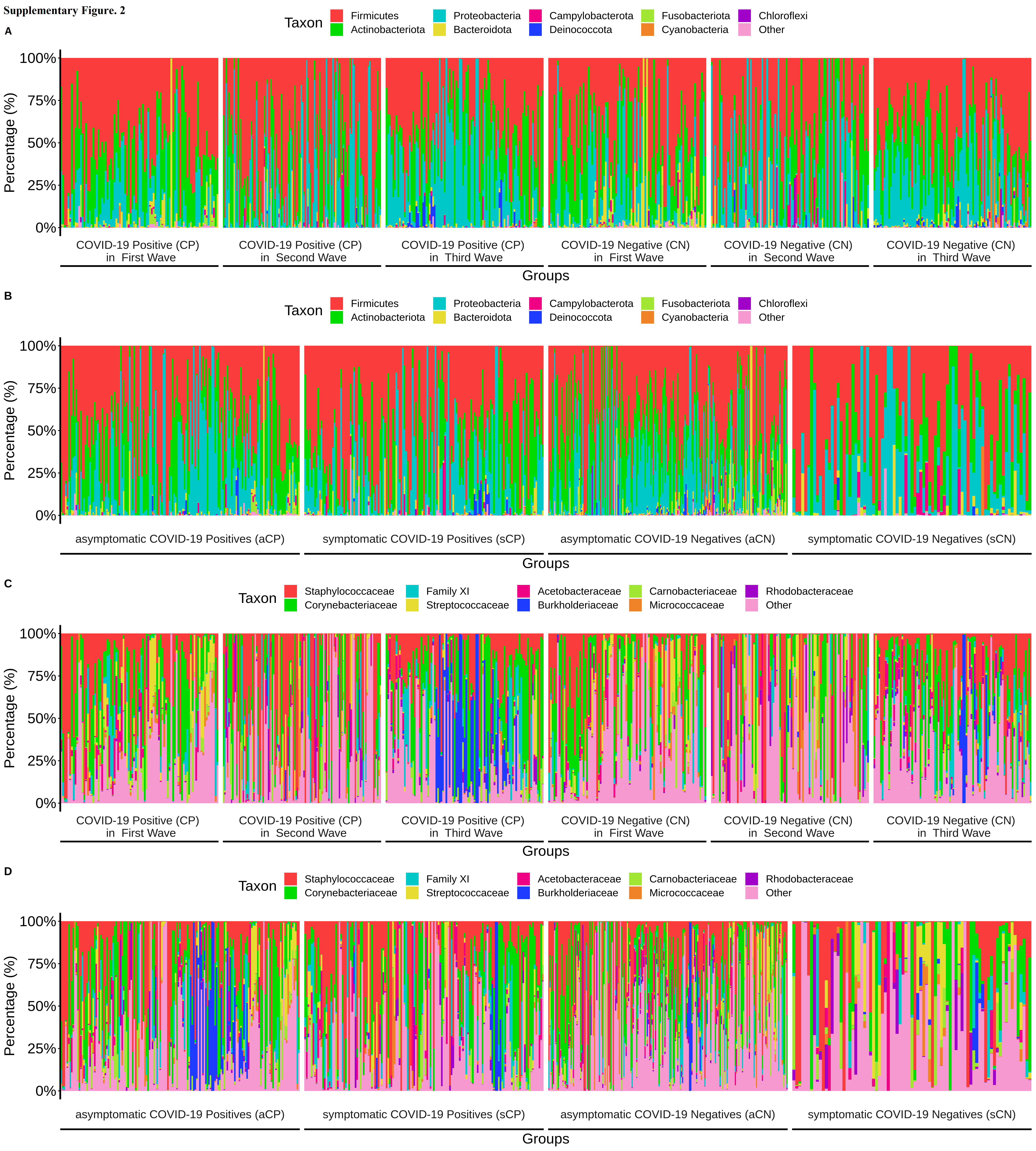

Supplement: Supplementary Figure S2 — Stacked bar-plot representing the distribution of the bacterial taxonomies across samples. Distribution of bacteria at (A) phylum and (B) family level in CP & CN samples across the three COVID-19 waves. Distribution of bacteria at (C) phylum and (D) family level between the four sample sub-groups: aCP, sCP, aCN & sCN. [file Image_2.TIF]

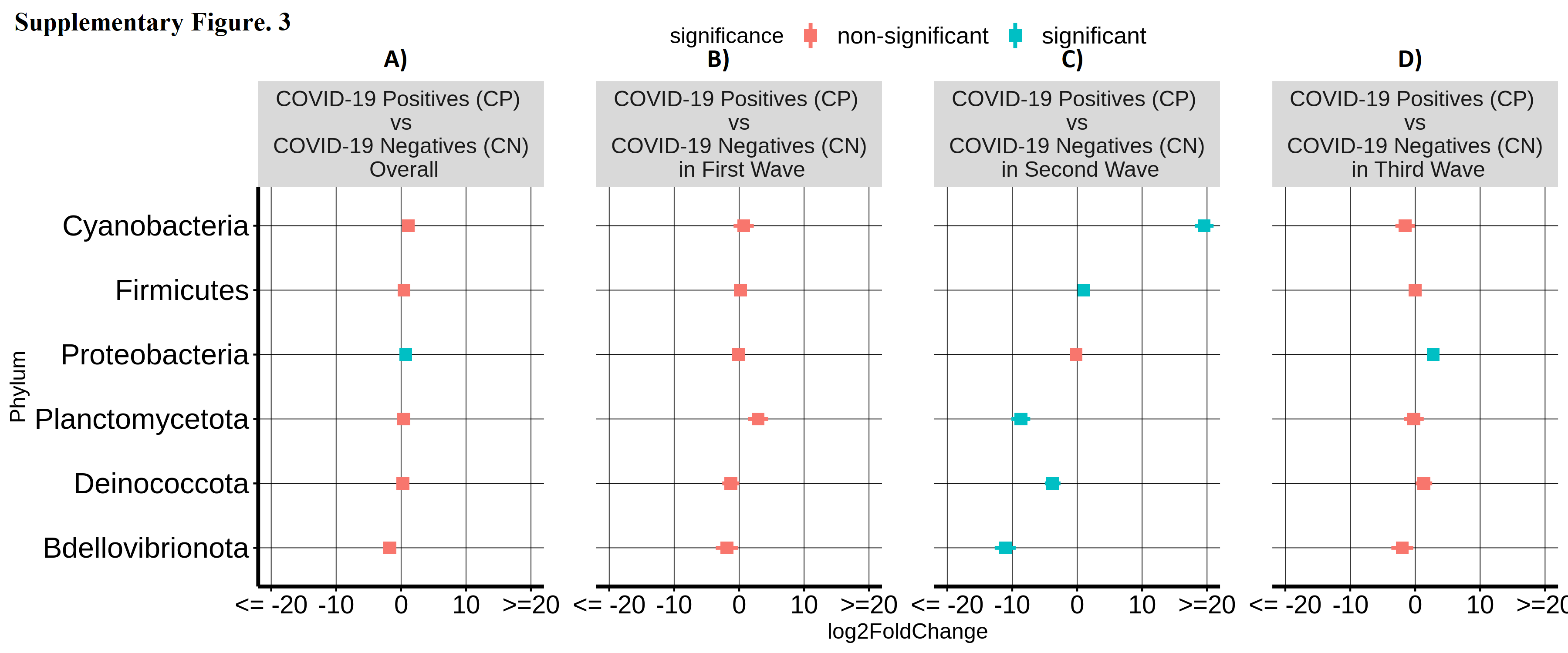

Supplement: Supplementary Figure S3 — Differential abundance of bacterial phylum between the CP & CN samples. The log2fold change in the mean abundance (along with whiskers representing standard errors) of a bacterial phylum in CP with respect to CN is depicted in (A) all the analysed samples (overall) as well as in (B-D) each of the three COVID-19 waves. Significantly different abundance (q-value < 0.05) is indicated with blue colour. [file Image_3.TIF]

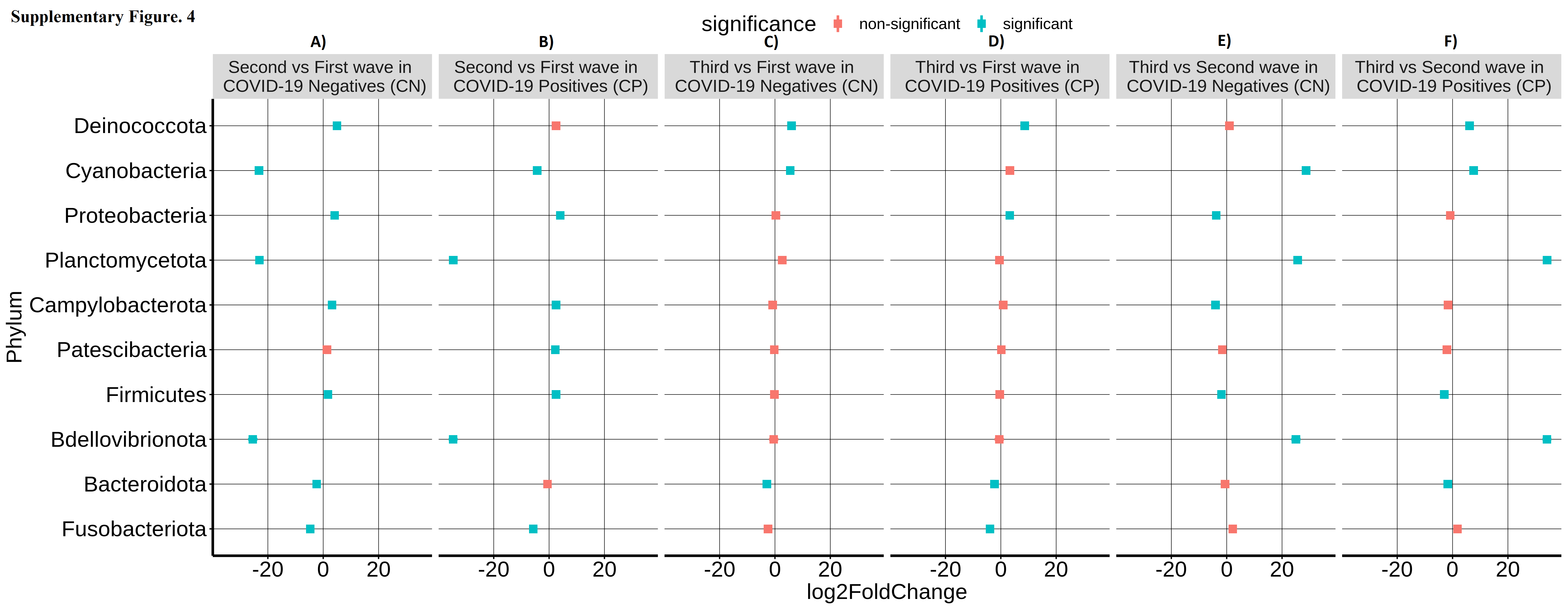

Supplement: Supplementary Figure S4 — Differential abundance of bacterial phylum between the CP & CN samples across the three COVID-19 waves. The log2fold change in the mean abundance (along with whiskers representing standard errors) of a bacterial phylum in the second wave with respect to the first wave in (A) CN samples, (B) CP samples; third wave with respect to the first wave in (C) CN samples, (D) CP samples; third wave with respect to the second wave in (E) CN samples, (F) CP samples. Significantly different abundance (q-value < 0.05) is indicated with blue colour. [file Image_4.TIF]

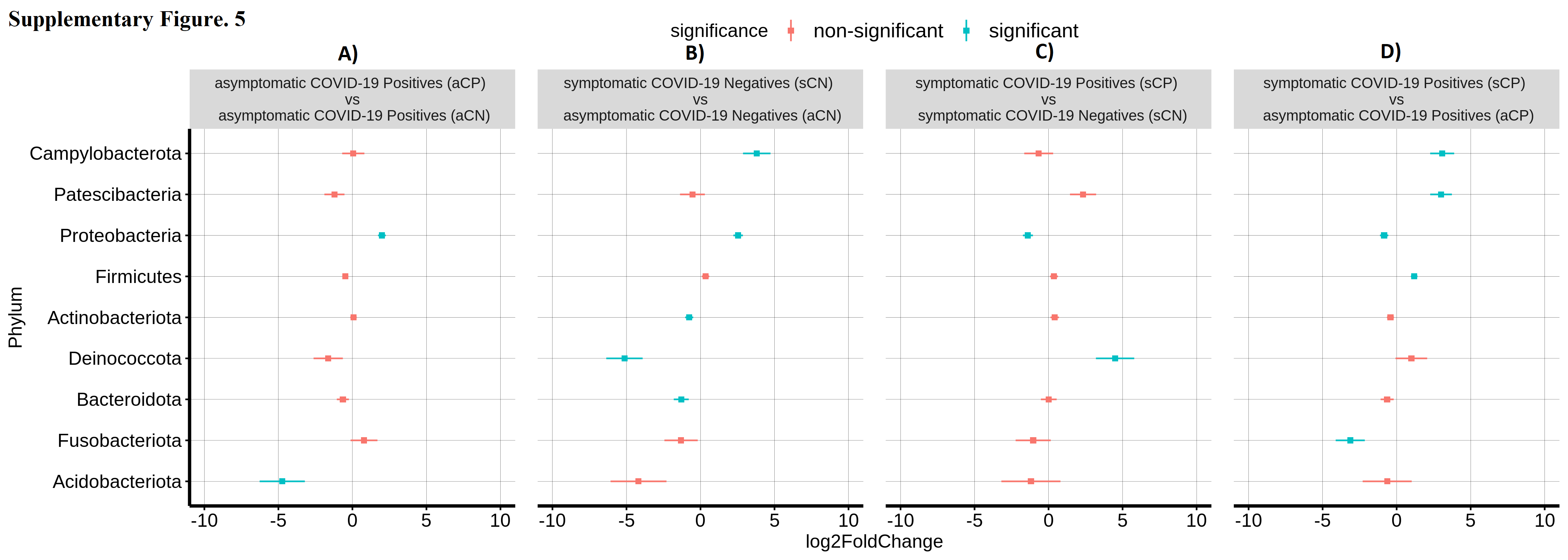

Supplement: Supplementary Figure S5 — Differential abundance of bacterial phylum between the four sub-group of samples - aCP, sCP, aCN & sCN. The log2fold change in the mean abundance (along with whiskers representing standard errors) of a bacterial phylum in (A) aCP with respect to aCN, (B) sCN with respect to aCN, (C) sCP with respect to sCN, and (D) sCP with respect to aCP is depicted. Significantly different abundance (q-value < 0.05) is indicated with blue colour. [file Image_5.TIF]

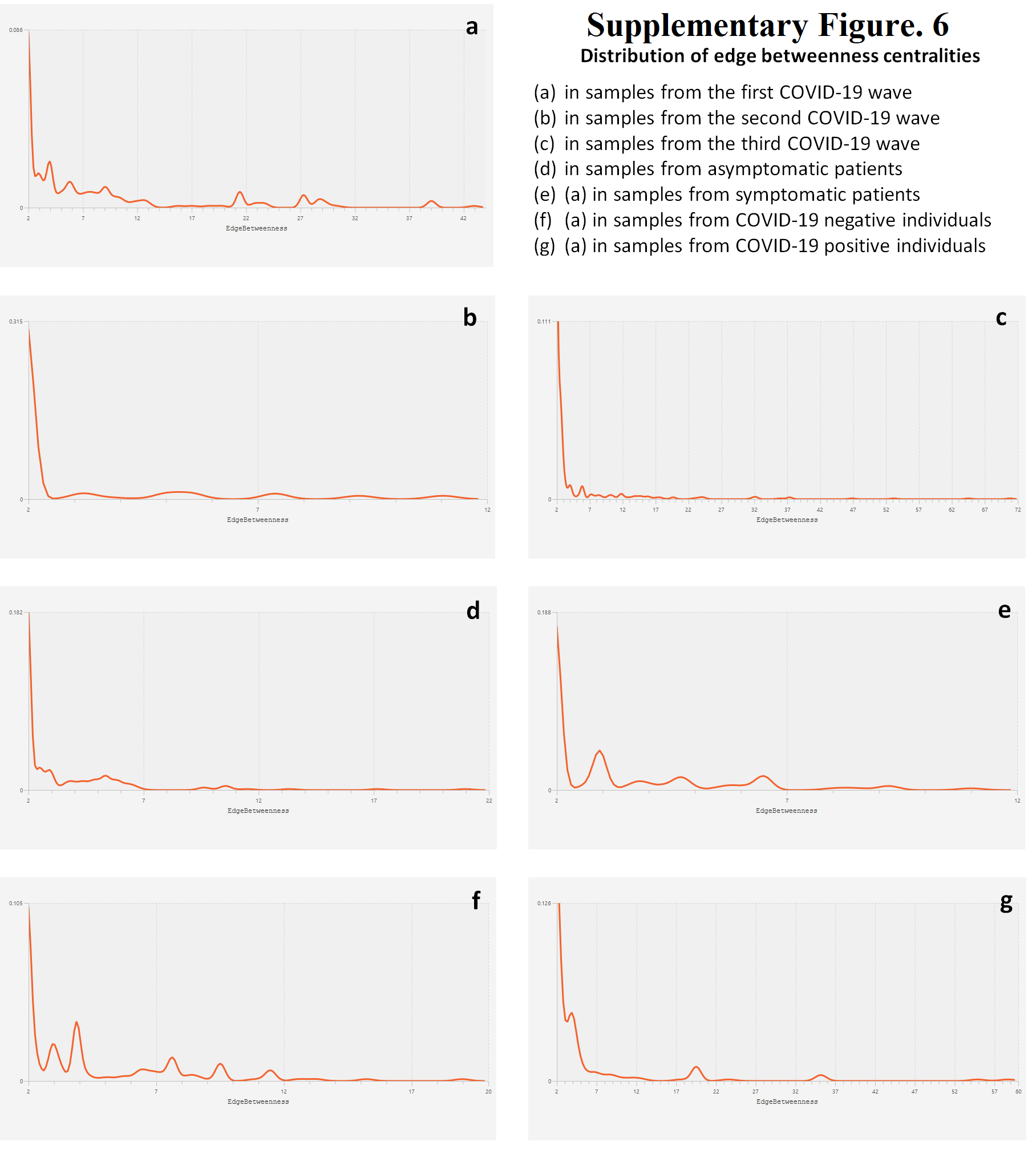

Supplement: Supplementary Figure S6 — Distribution of betweenness centralities of the nodes (microbes) in each of the analysed microbial association network. [file Image_6.TIF]

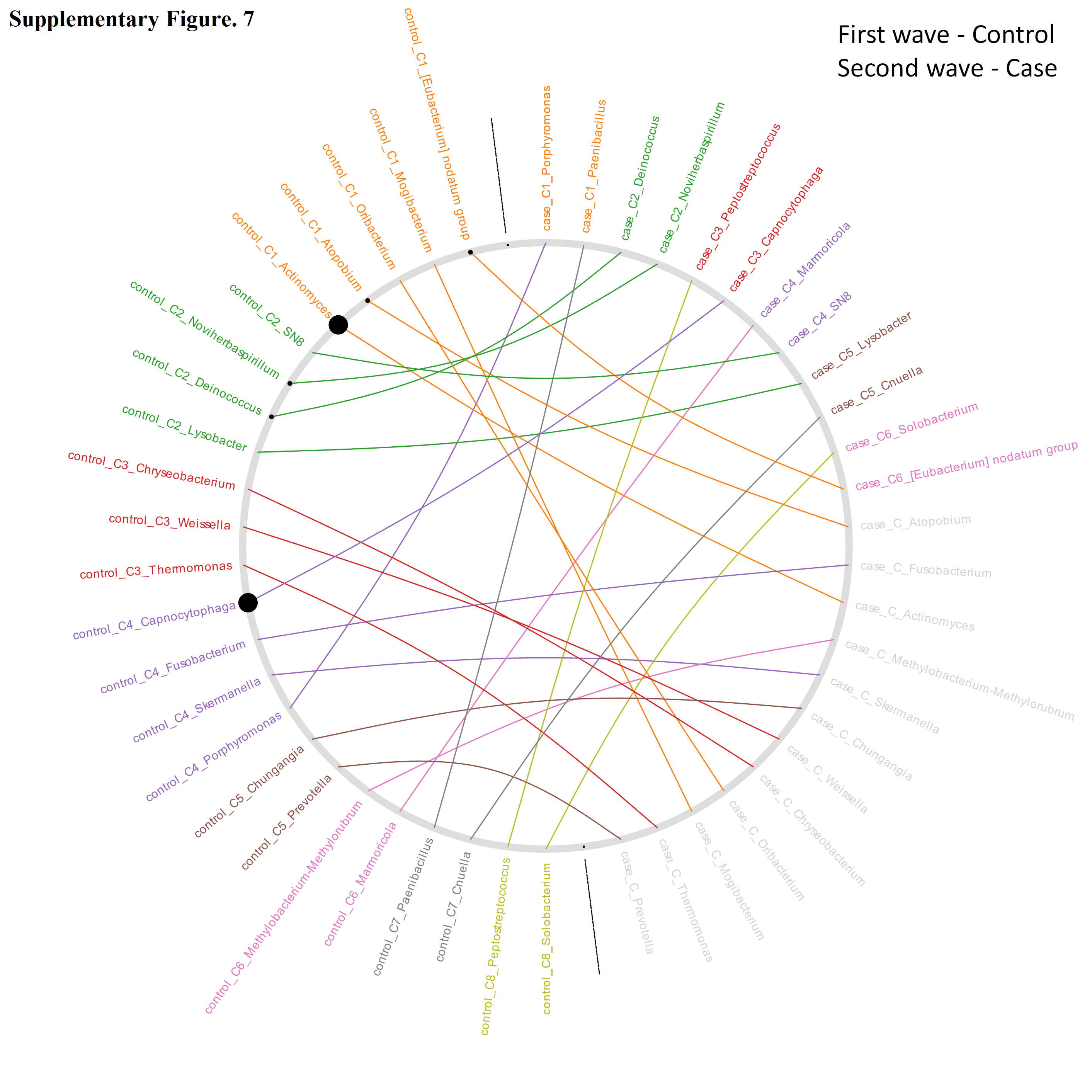

Supplement: Supplementary FIGURE S7 — The changes in community structure (community shuffling) between the microbial association networks corresponding to the first COVID-19 wave - ‘control’ and the second COVID-19 wave - ‘case’ networks. Nodes belonging to the ‘control’ and ‘case’ networks are plotted along the left half and right half of the circular frame. Same node (microbe) in the two network is connected by an edge for easy viewing of the community shuffling. Node labels are coloured (at random) based on sub-network/ community affiliations. Greyed out node labels indicate that the node does not interact directly with the common sub-network. The node sizes are proportional to the betweenness centrality measure of the node in the corresponding network. [file Image_7.TIF]

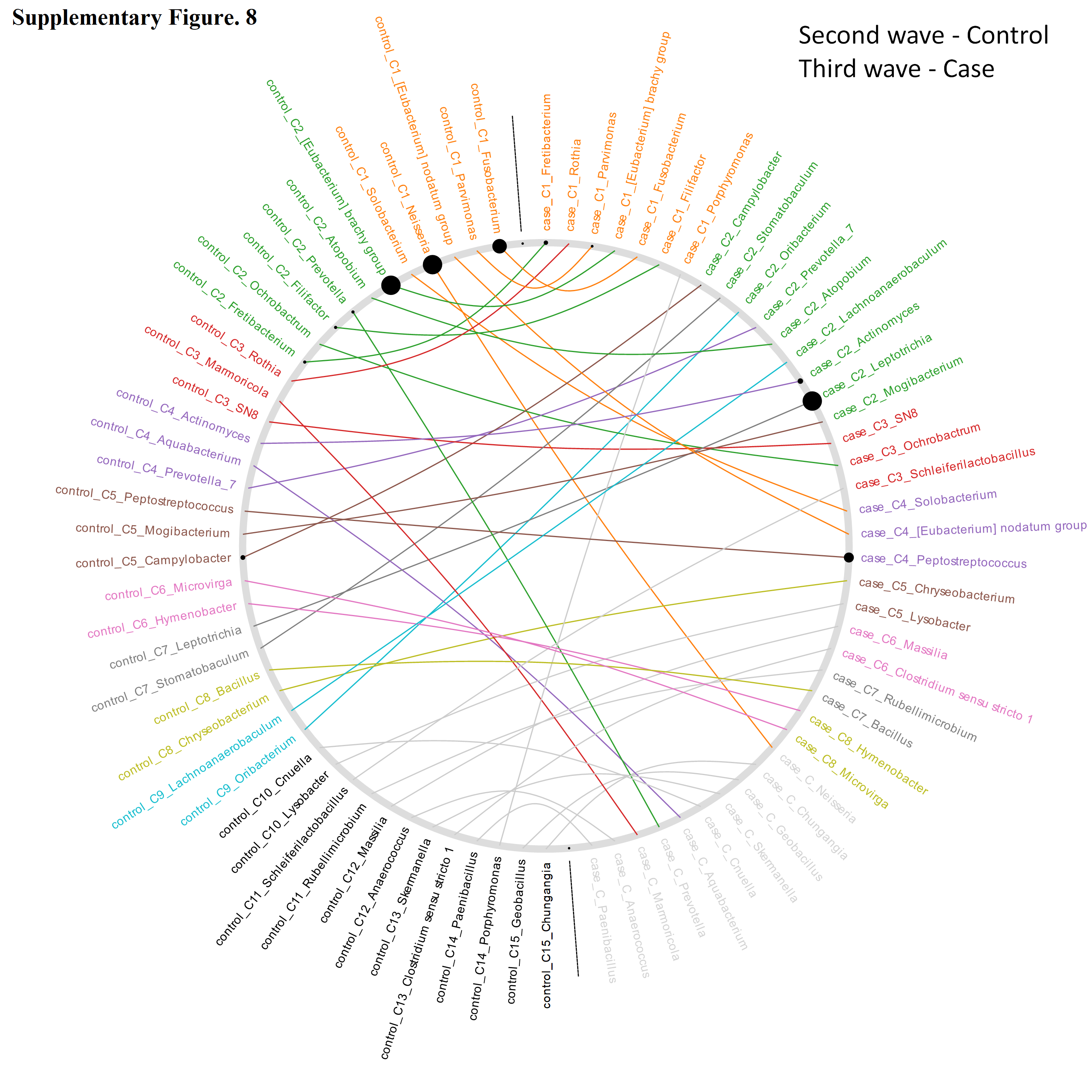

Supplement: Supplementary FIGURE S8 — The changes in community structure (community shuffling) between the microbial association networks corresponding to the second COVID-19 wave - ‘control’ and the third COVID-19 wave - ‘case’ networks. Nodes belonging to the ‘control’ and ‘case’ networks are plotted along the left half and right half of the circular frame. Same node (microbe) in the two network is connected by an edge for easy viewing of the community shuffling. Node labels are coloured (at random) based on sub-network/ community affiliations. Greyed out node labels indicate that the node does not interact directly with the common sub-network. The node sizes are proportional to the betweenness entrality measure of the node in the corresponding network. [file Image_8.TIF]

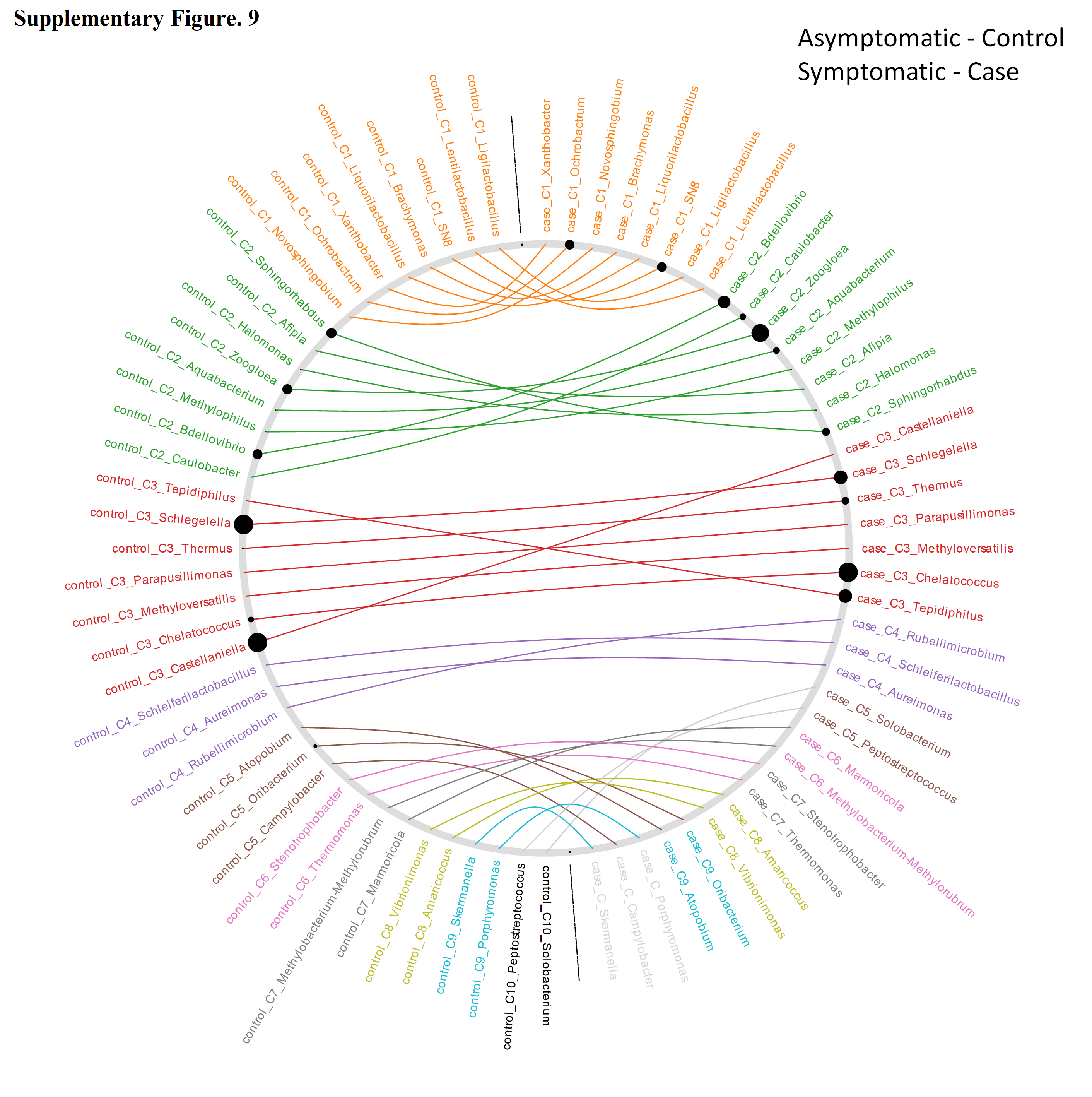

Supplement: Supplementary FIGURE S9 — The changes in community structure (community shuffling) between the microbial association networks corresponding to the samples from asymptomatic individuals - ‘control’ and the samples from symptomatic individuals - ‘case’ networks. Nodes belonging to the ‘control’ and ‘case’ networks are plotted along the left half and right half of the circular frame. Same node (microbe) in the two network is connected by an edge for easy viewing of the community shuffling. Node labels are coloured (at random) based on sub-network/ community affiliations. Greyed out node labels indicate that the node does not interact directly with the common sub-network. The node sizes are proportional to the betweenness centrality measure of the node in the corresponding network. [file Image_9.TIF]
